# YfiBNR Mediates Cyclic di-GMP Dependent Small Colony Variant Formation and Persistence in *Pseudomonas aeruginosa*

**DOI:** 10.1371/journal.ppat.1000804

**Published:** 2010-03-12

**Authors:** Jacob G. Malone, Tina Jaeger, Christian Spangler, Daniel Ritz, Anne Spang, Cécile Arrieumerlou, Volkhard Kaever, Regine Landmann, Urs Jenal

**Affiliations:** 1 Biozentrum, University of Basel, Basel, Switzerland; 2 Institute of Pharmacology, Hannover Medical School, Hannover, Germany; 3 Actelion Pharmaceuticals Ltd., Allschwil, Switzerland; 4 Department of Biomedicine, University Hospital, Basel, Switzerland; Yale University School of Medicine, United States of America

## Abstract

During long-term cystic fibrosis lung infections, *Pseudomonas aeruginosa* undergoes genetic adaptation resulting in progressively increased persistence and the generation of adaptive colony morphotypes. This includes small colony variants (SCVs), auto-aggregative, hyper-adherent cells whose appearance correlates with poor lung function and persistence of infection. The SCV morphotype is strongly linked to elevated levels of cyclic-di-GMP, a ubiquitous bacterial second messenger that regulates the transition between motile and sessile, cooperative lifestyles. A genetic screen in PA01 for SCV-related loci identified the *yfiBNR* operon, encoding a tripartite signaling module that regulates c-di-GMP levels in *P. aeruginosa*. Subsequent analysis determined that YfiN is a membrane-integral diguanylate cyclase whose activity is tightly controlled by YfiR, a small periplasmic protein, and the OmpA/Pal-like outer-membrane lipoprotein YfiB. Exopolysaccharide synthesis was identified as the principal downstream target for YfiBNR, with increased production of Pel and Psl exopolysaccharides responsible for many characteristic SCV behaviors. An *yfi*-dependent SCV was isolated from the sputum of a CF patient. Consequently, the effect of the SCV morphology on persistence of infection was analyzed *in vitro* and *in vivo* using the YfiN-mediated SCV as a representative strain. The SCV strain exhibited strong, exopolysaccharide-dependent resistance to nematode scavenging and macrophage phagocytosis. Furthermore, the SCV strain effectively persisted over many weeks in mouse infection models, despite exhibiting a marked fitness disadvantage *in vitro*. Exposure to sub-inhibitory concentrations of antibiotics significantly decreased both the number of suppressors arising, and the relative fitness disadvantage of the SCV mutant *in vitro*, suggesting that the SCV persistence phenotype may play a more important role during antimicrobial chemotherapy. This study establishes YfiBNR as an important player in *P. aeruginosa* persistence, and implicates a central role for c-di-GMP, and by extension the SCV phenotype in chronic infections.

## Author Summary

During long-term chronic infections of cystic fibrosis patients, *Pseudomonas aeruginosa* adapts to the lung environment, generating various different morphotypes including small colony variants (SCVs), small, strongly adherent colonies whose appearance correlates with persistence of infection. The SCV morphology is strongly associated with increased levels of the signaling molecule cyclic di-GMP. In this study we investigated the connection between cyclic di-GMP, SCV and persistence of infection. Following a genetic screen for mutants that displayed SCV morphologies, we identified and characterized the YfiBNR system. YfiN is a membrane-bound cyclic di-GMP producing enzyme, whose activity is tightly controlled by YfiR and YfiB. Cyclic di-GMP produced by YfiN boosts exopolysaccharide synthesis, generating an SCV morphotype upon YfiR-mediated release of YfiN repression. The resulting YfiN-mediated SCV morphotype is highly resistant to macrophage phagocytosis *in vitro*, suggesting a role for the SCV phenotype in immune system evasion. Consistent with this, YfiN de-repression increased the persistence of *P. aeruginosa* in long-term infections in a mouse model. The observation that the addition of antibiotics decreased the number of suppressors, and the relative fitness disadvantage of the YfiN-mediated SCV morphotype in liquid culture, suggested that SCV-mediated persistence might be favored during antimicrobial chemotherapy.

## Introduction

Many bacterial pathogens are able to establish long-term chronic infections within their respective hosts. The strategies and mechanisms employed by pathogens to persist against attacks by the host immune system and the action of antimicrobial substances are still relatively poorly understood. An appreciation of these processes is needed to develop strategies that help to avoid complications with antibiotic resistance development and infection relapses associated with prolonged colonization of host tissue. The opportunistic pathogen *Pseudomonas aeruginosa* is responsible for chronic infections in the airways of cystic fibrosis (CF) patients and for much of the associated morbidity and mortality [1]. During long-term lung colonization, *P. aeruginosa* undergoes phenotypic and genetic adaptation resulting in the progressive loss of virulence and the development of increased persistence [2]. As a manifestation of this process, distinct adaptive colony morphotypes of *P. aeruginosa* appear in CF sputum sample isolates. These include mucoid colonies, which overproduce the exopolysaccharide alginate [1], and small colony variants (SCVs), slow-growing isolates characterized by several phenotypes including auto-aggregation, attachment to surfaces [3] and enhanced exopolysaccharide production [3],[4]. The appearance of *P. aeruginosa* SCVs in CF sputum samples correlates with antibiotic resistance [5],[6], poor lung function, and persistence [7]. Likewise, *Burkholderia cepacia* SCVs are associated with increased serum resistance and fatal systemic infections post lung transplantation [8]. These studies suggest that during the course of chronic lung infections, SCVs are selected for due to a fitness advantage in this unique environment, and that they might play an important role in the pathogenesis of *P. aeruginosa* lung infections.

Following early indications [9],[10], evidence has accumulated that the SCV morphotype is strongly linked to the second messenger cyclic-di-GMP (c-di-GMP) [11]–[13]. A recent study of CF patients showed clear links between c-di-GMP-related systems, and hence SCV, and chronic lung infection [2]. C-di-GMP is an ubiquitous bacterial second messenger that regulates the sessile-motile transition in a wide range of species [14],[15]. C-di-GMP is produced from GTP by di-guanylate cyclases (DGC), and degraded to pGpG by phosphodiesterases (PDE) [16]. These enzymes contain the conserved GGDEF and EAL domains, respectively [17]–[20]. GGDEF domains that harbor enzymatic activity generally contain two conserved motifs; the catalytic active (A) site, and the regulatory (I) site, required for non-competitive product inhibition [21]. In general, elevated levels of c-di-GMP are associated with a sessile, cooperative lifestyle. Phenotypes expressed in this state include biofilm formation [22],[23], enhanced exopolysaccharide production [24],[25], expression and production of attachment factors [9],[26], loss of various forms of motility [27],[28], and reduced virulence [29],[30]. Conversely, low levels of c-di-GMP promote a unicellular, free-swimming lifestyle.

The c-di-GMP regulatory system in *P. aeruginosa* is highly complex, with 33 predicted GGDEF and 17 EAL domain-containing proteins [15],[29]. These proteins are thought to modulate the intracellular c-di-GMP level and hence regulate the production of numerous outputs. These include the exopolysaccharides Pel, Psl and alginate [25],[31],[32], fimbrial adhesins [26], virulence and cytotoxicity factors [29],[33], pili [27],[34],[35] and flagella function [36]. Regulation of these output systems has been observed at the transcriptional [32] and post-translational levels [25],[31]. To date, the biological roles and mechanisms of action of several *P. aeruginosa* c-di-GMP signaling systems have been investigated. These include the Wsp system [12],[37],[38], comprising a Che-like chemosensory system with a DGC response-regulator output, WspR [12],[37]. Over-activation of WspR results in an SCV phenotype [9],[12], displaying strong attachment and increased expression of the *pel* and *psl* exopolysaccharide operons [12],[13]. Sad/RocARS [26],[33] is a two-component signaling system (RocS1) with two output proteins; a transcriptional regulator (RocA1) and an EAL domain containing protein (RocR). Sad/RocARS is required for biofilm maturation [33] and regulates *cup* fimbriae expression [26]. This system is further implicated in pathogenicity, with links to cyanide-mediated toxicity [39] and type-III secretion [33]. The transition between reversible and irreversible attachment in *P. aeruginosa* is regulated by the putative hydrolase SadB [40], which promotes Pel exopolysaccharide production and represses swarming motility [40],[41]. These processes are similarly co-regulated by the opposing enzymatic activities of SadC, a membrane-bound DGC [36] and the PDE BifA [42]. In addition, c-di-GMP has been associated with the response of *P. aeruginosa* to antibiotic [10],[43],[44] and detergent-mediated [45] stress, and with type IV pili production and control [27],[34].

To investigate the role of c-di-GMP in the generation of the *P. aeruginosa* SCV phenotype, we undertook a transposon mutagenesis screen in strain PA01 for genes whose disruption produced autoaggregative, Congo Red binding colonies. Three independent transposon insertions were found in *yfiR*, the first gene of the *yfiBNR* operon, which is highly conserved in many γ-proteobacteria. Here we characterize the *yfiBNR* genes, and report that this conserved system represents a tripartite signaling module that regulates c-di-GMP levels in *P. aeruginosa* in response to as-yet unknown environmental signals. YfiN acts as a membrane-integral DGC whose activity is tightly controlled by YfiR, a small periplasmic protein, and the OmpA/Pal-like outer-membrane lipoprotein YfiB. The primary downstream target for the cyclic-di-GMP produced by YfiBNR was identified as the Pel exopolysaccharide system, with transcription of *pel* genes being directly proportional to the inferred output level of YfiN. Disruption of the tight YfiN regulation via deletion of *yfiR* resulted in the generation of an SCV morphology in PA01. Consequently, the Δ*yfiR* genotype was used as a representative SCV in cell culture assays and in a mouse model of persistent infection. Compared to PA01, the Δ*yfiR* mutant exhibited strong, exopolysaccharide-dependent resistance to macrophage phagocytosis. In addition, the SCV phenotype was shown to effectively persist in a subcutaneous catheter infection model. Together this establishes the YfiBNR module as an important player in the persistence of *P. aeruginosa* and implicates a role for c-di-GMP in chronic infections.

## Materials and Methods

### Strains and growth conditions

Strains and plasmids used in this study are listed in Table S1. Primers are listed in Table S2. Unless otherwise stated, *P. aeruginosa* PA01 and all *E. coli* strains were grown at 37°C in Luria Bertani (LB) medium [46], solidified with 1.3% agar where appropriate. For *P. aeruginosa*, gentamycin was used at 30 µg/ml (*E. coli* 20 µg/ml), tetracycline at 50-100 µg/ml (*E. coli* 12.5 µg/ml), streptomycin at 200 µg/ml (*E. coli* 50 µg/ml), and carbenicillin at 200 µg/ml. For *E. coli*, kanamycin was used at 30 µg/ml and ampicillin at 100 µg/ml. Where required, chloramphenicol was added to conjugation plates at 10 µg/ml. Congo Red dye was added to a final concentration 0.04%. For inducible plasmids, vanillate was added to a final concentration 1.0 mM, arabinose to 0.2% and IPTG to 0.5 mM, as appropriate. J774 macrophages were cultured in 150 µl RPMI medium (Sigma) supplemented with 2% Glutamine and 2% FCS, and incubated at 37°C, 5% CO_2_.

### Molecular biology procedures

Cloning was carried out in accordance with standard molecular biology techniques. pBBR5-*yfiR* was produced by ligation of the *yfiR* PCR fragment (amplified with primers A and B, from PA01 genomic DNA), between the *Hind*III and *Bam*HI sites of pBBR-MCS5 [47]. pME-*araC* was constructed by ligation of the *araC*-*pBAD* promoter fragment of pBAD18s [48] between the *Bam*HI and *Eco*RI sites of pME6032. pME-*araC-yfiN* was produced by ligation of the *yfiN* PCR fragment (amplified with primers C and D, from PA01 genomic DNA), between the *Sac*I and *Bgl*II sites of pME-*araC*. Truncated and non-truncated *yfiR-phoA* fusion plasmids, and pME-*yfiR-cherry* were produced by ligation of SOE-PCR/PCR fragments generated with primers F-J, BJ and BK between the *Eco*RI and *Xho*I sites of pME-*araC*, and the *Eco*RI and *Bgl*II sites of pME6032 respectively. The *yfiBNR* complementation vectors were constructed by ligation of the relevant *yfiBNR* PCR fragments (amplified with primers A, D, and E from PA01 and Δ*yfiR* genomic DNA) between the *Hin*dIII and *Bam*HI sites of pUC18T-mini-Tn*7*T-Gm [49]. A and I site *yfiN* mutants were constructed via SOE PCR [50], from template PCR fragments amplified with primers A, D, E, and K-N. The plasmid pME6032-*yfiB* was constructed by ligation of the *yfiB* PCR fragment (amplified with primers E and O, from PA01 genomic DNA), between the *Eco*RI and *Bam*HI sites of pME6032 [51]. Plasmid pBBR4-*wspR* was constructed by ligation of the *Eco*RI*-Hin*dIII *wspR* fragment of plasmid pWspR12 [52] between the *Eco*RI and *Hin*dIII sites of pBBR-MCS4 [47].

To produce a chromosomal M2-tagged copy of *yfiR*, the PCR fragment amplified with primers A and E was ligated between the *Hin*dIII and *Bam*HI sites of pMR20 [53] and *E. coli* DY330 [54] was transformed with the resulting plasmid. The PCR fragment amplified with primers P and Q from plasmid pSUB11 [55] was then used to produce an *yfiR*-M2 fusion in pMR20 by the method described by Yu et al. [54]. The *yfiR*-M2 fusion was ligated into the *Hin*dIII and *Bam*HI sites of pUC18T-mini-Tn7T-Gm. The PCR fragment amplified with primers R and S was used to produce pAD6-Ω from plasmid pAD6 (A. Dürig, unpublished), by the method described in [54].

The plasmids pET42b-*yfiN* and *yfiB* were constructed by ligation of the relevant PCR fragments (amplified with primers T-W, from PA01 genomic DNA), between the *Xho*I and *Nde*I sites of pET42b (Novagen). Bacterial two-hybrid vectors were constructed by ligation of the relevant PCR fragments (amplified with primers D, E, and X-AK from PA01 genomic DNA) between the *Xba*I and *Bam*HI sites of pUT18C and pKT25, and the *Hin*dIII and *Bam*HI sites of pUT18 [56].

Plasmid pME6032*-luxCDABE* was constructed by ligation of the *Eco*RI*-Bam*HI fragment of plasmid pSB417 [57] between the *Eco*RI and *Bam*HI sites of pME6032. Lux fusions with the *cupA* and *pel* promoters were then constructed by ligation of *EcoRI-*excised promoter fragments from plasmids pMPFCAL [58] and p*-pelA-lacZ* [59] respectively into the *Eco*RI site of pME6032*-luxCDABE*. Lux fusions with the *cupB*, *cupC*, *psl* and *yfi* promoters were constructed by ligation of the relevant PCR fragments (amplified with primers AL-AS, from PA01 genomic DNA), between the *Xho*I and *Eco*RI sites of pME6032*-luxCDABE*.

### Transduction with phage E79tv2

E79tv2 [60] transducing lysates were prepared as follows: 100 µl of a donor overnight culture were mixed with 100 µl of wild type phage (app. 10^7^ to 10^5^ PFU/ml), incubated for 10 min at 37°C and mixed with 3 ml top-agar. The mixture was poured onto LB plates and incubated at 37°C overnight. Top-agar with semi-confluent plaques was then scraped from the plates and re-suspended in 2 ml of TNM buffer (10 mM Tris HCl [pH 7.4], 150 mM NaCl, 10 mM MgSO_4_), vortexed and centrifuged at 20,000 g for 10 min, the supernatant containing the transducing phage was then transferred into a glass storage vial. For transduction, 500 µl of an overnight culture of recipient strain was re-suspended in 500 µl TNM buffer, mixed with 500 µl of UV-attenuated transducing lysate (5×10^8^ PFU/ml, 15 sec. UV Stratalinker 2400) and incubated at 37°C for 15 min. Non-adsorbed phage was removed by washing twice in TNM buffer and samples were plated onto selective media.

### Transposon mutagenesis

The plasmid pALMAR3 was introduced into PA01 via biparental mating with *E. coli* S17-1. Mariner transposon insertions yielding an SCV morphology were selected on LB agar containing tetracycline, chloramphenicol and Congo Red, and restreaked onto fresh plates. The location of the transposon in each SCV strain was determined by semi-random PCR [61], using primers AT-AW.

### Deletion of the *yfiBNR* genes

The strains PA01 Δ*yfiNR* and Δ*yfiBNR* were constructed via an adaptation of the protocol described elsewhere [62]. Briefly, deletion constructs were produced by SOE-PCR using primers AX-BE, containing homologous flanking regions to the target genes and FLP-excisable gentamycin cassettes. These constructs were ligated into pEX18Ap between *Hin*dIII and *Kpn*I. The resulting vectors were then used to delete *yfiNR*/*BNR* as described in [62]. PA01 Δ*yfiR* was deleted by two-step allelic exchange. A deletion construct containing the flanking regions of *yfiR* was produced by SOE-PCR using primers BF-BI and ligated between *Hin*dIII and *Kpn*I of pEX18Ap. The resulting vector was then transformed into PA01. Following transformation, single crossovers were selected on carbenicillin and restreaked. Counterselection on 5% sucrose plates was used to force the resolution of double crossovers. In all cases, deletion strains were confirmed by colony PCR.

### Attachment assays

Assays were adapted from [63]. 96 well plates containing 150 µl LB medium/well were inoculated with single colonies using sterile toothpicks, and incubated overnight at 37°C without shaking. Plates were washed three times with distilled water. Remaining cell material was then stained with 0.1% Crystal Violet solution (5% methanol, 5% isopropanol) before further washing to remove excess dye. Crystal Violet was re-dissolved in 20% acetic acid solution and absorbance measured at 600 nm. Assays were performed with 6 wells/strain and repeated independently at least once in each case.

### Motility assays

Swarm agar was based on TB and solidified with 0.5% agar. After solidification, plates were dried overnight and then inoculated on the surface with colonies re-suspended in LB. Plates were incubated overnight at 37°C. Twitching motility was assayed using TB plates containing 1% agar. Samples were stabbed through the surface using toothpicks and plates incubated overnight at 37°C. Twitching cells were visualized by staining with 1% Crystal Violet following removal of the agar. Swimming motility was assayed using TB plates containing 0.3% agar. Colonies were stabbed into the agar using toothpicks and plates incubated overnight at 37°C. Assays were repeated three times independently.

### Purification of N-terminal truncated YfiN and YfiB-His_6_ and production of antisera

200 ml of overnight cultures of BL21 pET42b-*yfiN/yfiB* were used to inoculate 2L LB medium supplemented with kanamycin. Cultures were grown for 2.5 hours at 37°C with shaking, *His_6_-yfiN/B* expression was induced with 100 µM IPTG, and cultures incubated for a further 2.5 hours at 30°C with shaking. Cells were harvested by centrifugation and lysed with 15 ml lysis buffer (250 mM NaCl, 10 mM Tris pH 8.0, 6.0 M urea). Insoluble cell debris was harvested by centrifugation (30,000xg, 15 min, 4°C), and truncated-YfiN-His_6_ and YfiB-His_6_ were purified from the supernatant by nickel-NTA (BioRad) affinity chromatography. Following binding to 0.5 ml nickel-NTA (4°C, 30 min, shaking) proteins were eluted with a stepwise increase in imidazole concentration, with both proteins eluting in 200 mM imidazole. Native truncated-YfiN-His_6_ was purified as described, but without 6.0 M urea and with the addition of 10% glycerol to the lysis buffer. A French Press was used to lyse the sample in place of urea. Purified proteins were separated on preparative SDS gels, stained with 4 M KCl, excised and sent to Laboratoire d'Hormonologie, Marloie, Belgium for polyclonal antisera production. Protein concentrations were assayed with a Protein Assay kit (BioRad).

### Immunoblot analysis

Cell lysates from overnight cultures were separated on 15% Tris-HCl gels and blotted onto polyvinylidene difluoride (PVDF) membranes (Millipore). After overnight incubation in blocking solution (1 x PBS pH 7.4, 0.01% Tween20, 5% milk powder), proteins were detected with 1/5000 (α-M2, α-YfiB) or 1/500 (α-YfiN) specific antiserum and 1/10,000 rabbit anti-mouse (α-M2) or swine anti-rabbit (α-YfiB, α-YfiN) secondary antibody (DakoCytomation). Bound antibodies were visualized with ECL chemiluminescent detection reagent (Perkin-Elmer).

### Diguanylate cyclase (DGC) activity assay

DGC activity was assayed as described previously [18],[21]. Assays were run in 50 µl running buffer containing approximately 25 µg purified truncated-YfiN-His_6_ and started by the addition of (final concentration) 100 µM GTP [18.5 kBq α33P-GTP] (Amersham Biosciences). Samples were removed at regular intervals and the reaction stopped with (final concentration) 250 mM EDTA. Purified DgcA [21] was included as a positive control.

### Nucleotide extraction and analysis

Following protein purification, 100 µl of the truncated-YfiN-His_6_ elution fraction was mixed with 200 µl of 0.5 M formic acid, and nucleotides were extracted for 10 min at 4°C. Insoluble components were then pelleted, and the supernatant directly analyzed by chromatography after [21]. Nucleotides were extracted and separated on a 125/4 Nucleosil 4000-1 polyethyleneimine column (Macherey-Nagel) using the SMART System (Amersham Biosciences). The nucleotide peak corresponding to c-di-GMP was verified by co-elution with a chemically synthesized c-di-GMP standard.

### Quantitation of cyclic-di-GMP by mass spectrometry

Log phase growing cultures of PA01 strains were cooled rapidly in iced water and 5-10 ml of cells were concentrated by centrifugation (5,300xg, 10 min, 4°C). Extracts were prepared essentially as described in [64]. C-di-GMP levels were measured by liquid chromatography-tandem mass spectrometry on an API 3000 triple quadrupole mass spectrometer (Applied Biosystems Inc, Foster City, CA, USA) coupled with a Series 200 HPLC System (Perkin Elmer Instruments, Norwalk, CT, USA). C-di-GMP was detected via selected reaction monitoring (SRM) in positive ionization mode. Liquid chromatography separation was achieved on a reversed-phase column using an ammonium acetate-methanol gradient (retention time for c-di-GMP: 8.6 min). Details of this method will be described elsewhere (Spangler C. *et al.*, manuscript in preparation). Following nucleotide extraction, the pellet was dissolved in 800 µl 0.1 M NaOH by heating for 15 min at 95°C. The protein content of each pellet was then determined with a Protein Assay kit (BioRad). Measurements were repeated at least in triplicate and values were expressed as pmol c-di-GMP per mg protein.

### *In-vivo* crosslinking assays

Overnight cultures of PA01 strains were washed once and re-suspended in 1 volume PBS. An aliquot was removed, and crosslinking started by the addition of 1% formaldehyde. Aliquots were removed after 20 minutes crosslinking at room temperature, and washed once to remove formaldehyde. Benzon nuclease was added to digest DNA (5 U, 30 min, RT) and samples were boiled (20 min, 95°C) where required. Samples were subsequently analyzed by Western blotting.

### Subcellular localization of proteins

For the fractionation of soluble and membrane proteins, an overnight culture of PA01::*yfiR-M2* was re-suspended in 0.2 volumes lysis buffer (20 mM Tris pH 8.0, 250 mM NaCl, protease inhibitor cocktail (Roche)). Cells were lysed by French Press and centrifuged to remove cell debris (10,000 g, 1 hour, 4°C). The soluble and insoluble cell fractions were then separated by ultracentrifugation (100,000 g, 3 hours, 4°C). Samples were subsequently analyzed by immunoblotting. For the fractionation of periplasmic proteins, overnight cultures were resuspended in 0.125 volumes osmotic shock buffer (50 mM Tris pH 8.0, 20% sucrose, 2 mM EDTA) and incubated at room temperature for 30 to 60 min, depending on the strain. Samples were centrifuged (15,000 g, 10 minutes, 4°C) and the supernatant and pellet collected. The pellet fraction was washed twice and resuspended in PBS. The supernatant (periplasmic fraction) was either subjected to alkaline phosphatase assays directly, or proteins were precipitated with 5% TCA, washed twice with di-ethyl ether and resuspended in PBS before immunoblot analysis.

### Alkaline phosphatase assay

Alkaline phosphatase activity was assayed for YfiR-PhoA protein fusions by measuring the rate of p-nitrophenyl-phosphate hydrolysis according to the method described in [65]. Assays were carried out in triplicate for each protein fusion and the experiment was repeated independently.

### Bacterial two-hybrid

Bacterial two-hybrid assays were carried out after [66]. *E. coli* MM337 cells were freshly transformed with constructs containing potential interaction partners. Transformants were then streaked onto MacConkey base and M9 plates, supplemented with 0.5% maltose, kanamycin and ampicillin. Plates were incubated for two days at 30°C and positive interactions distinguished by red coloration on MacConkey and growth on M9 plates.

### Luminescence assay for promoter activity

Strains containing pME6032*-promoter-lux* fusion constructs were streaked onto LB plates containing tetracycline, incubated overnight and the resulting colonies used to inoculate LB cultures. These cultures were grown for 6 hours at 37°C and then two 150 µl aliquots were transferred to a black, clear-bottomed 96-well plate (Costar). Luminescence and OD_600_ were recorded for each sample using a Synergy 2 plate reader (Biotek). Samples were repeated in triplicate for each promoter fusion and the assay repeated independently at least twice.

### Scanning electron microscopy

Cells were grown overnight at 37°C, in 2 ml LB in 24-well plates and in the presence of a hanging sterile glass slide. After growth, the glass slides were removed, rinsed gently with 1x PBS and fixed in 2.5% glutaraldehyde in 1x PBS for 2 h at RT. Glutaraldehyde was washed out with 1x PBS and subsequently with water, and the sample dehydrated with an acetone step gradient (30%, 50%, 70%, 90%, 100%; 10 min each). Samples were critical point-dried and sputter-coated with a 3–5 nm platinum layer. Micrographs were recorded on a Hitachi S-4800 field emission scanning electron microscope. Acceleration voltage was generally between 1.5 and 5 kV.

### Nematode absorption assays

100 µL drops of PA01 overnight cultures were dried onto M9 plates with 0.4% glucose, and the plates incubated overnight at 37 °C. Approximately 20 adult *Caenorhabditis elegans* were added to each plate, and plates were incubated at room temperature for 72 hours. For GFP labeling experiments, PA01 strains containing pAD6-Ω were used and incubated with *C. elegans* for 3 hours before imaging.

### Macrophage phagocytosis and NF-κB activation assays

J774 macrophages were incubated overnight in a black, clear-bottomed 96-well plate, at a concentration of 2×10^4^ cells/well. Lysotracker red dye (Invitrogen) was added to each well 30 minutes prior to infection. PA01 strains containing pAD6-Ω were grown in LB for 4 hours at 37°C and then used to infect the mature macrophages (final MOI 5). Samples were vortexed thoroughly at every stage of the preparation process to minimize the proportion of cell aggregates in the inoculum. After 2 hours incubation at 37°C, 5% CO_2_, macrophages were fixed by the addition of 4% PFA solution. Wells were washed with PBS and stained with Hoechst 33342, then subjected to analysis with an ImageXpress^MICRO^ microscope running MetaXpress 2.0 (Molecular Devices). For the NF-κB activation assay, the Lysotracker stain was omitted, and the macrophages permeabilised with Triton-X100 and stained with an anti-NF-κB p65 antibody (Santa Cruz Biotechnology) following infection. The Translocation enhanced analysis module of MetaXpress was used to calculate the ratio of cytosolic to nuclear p65 intensity at the single cell level. Six wells were inoculated per strain and each assay was repeated independently.

### Siderophore and pyocyanin production

Siderophore and pyocyanin levels were measured in the supernatants of overnight cultures grown in M9 plus 0.4% glucose and 0.2% casamino acids (Pyoverdin, Pyochelin) and LB (Pyocyanin) using the methods described in [67].

### LDH release assay for cytotoxicity

J774 macrophages were inoculated in a 24 well plate, at a concentration of 1×10^5^ cells/well and incubated overnight. Overnight cultures of PA01 strains were inoculated into LB, grown for 6 hours at 37°C and used to infect the mature macrophages (final MOI 10). After 3 hours incubation (37°C, 5% CO_2_), LDH release was measured with a CytoTox96 kit (Promega) according to the manufacturer's instructions. Values were calculated as a percentage of the positive control (+ 0.5% TritonX100), and each sample was repeated in triplicate.

### Infection models

C57BL/6 mice (10–14 weeks old) were obtained from RCC (Füllinsdorf, BL, Switzerland) and kept in the animal facility of the Department of Biomedicine, University Hospitals Basel; animal experimentation guidelines were followed in accordance with the regulations of Swiss veterinary law. Methods employed were approved by the review board of the Kantonales Veterinäramt Basel-Stadt (permit no. 1957). Mice were infected as described elsewhere [68], with minor modifications. Briefly, mice were anesthetized with 20 mg/kg Ketalar (Pfizer) and 4 mg/kg xylazinum (Graeub). A 3–4 mm incision was made 1–1.5 cm lateral to the spine, and a catheter segment (1 cm Vialon IV catheters, diameter of 2.1 mm; Becton Dickinson), was inserted subcutaneously. Next, 25 µl of pyrogen-free saline containing 10,000 cfu of PA01-Gm^R^ (wt), Δ*yfiR* or Δ*yfiBNR* bacteria grown to exponential phase in LB medium was injected into the beds of uncoated catheters, and the incision was closed with wound clips. Four and eight weeks after infection, mice were sacrificed and the catheter and surrounding tissue were aseptically removed and separated. Catheters were vortexed in saline, 0.15% EDTA, and 0.1% Triton X-100 and sonicated for 3 min at 130 W. Tissue samples were homogenized, dilutions from both preparations were plated onto LB agar plates, and colony-forming units were counted following overnight incubation at 37°C. For competition experiments, wt and Δ*yfiR* or Δ*yfiBNR* bacteria were injected at a 1∶1 ratio. Differences in colony morphology were used to distinguish between wild type and Δ*yfiR* mutant colonies. Statistically significant differences were determined using the Mann-Whitney test.

### Antibiotic survival assay

Glass tubes containing 4 ml LB and tobramycin (0–2.0 µg/ml) were inoculated with 10,000 cfu of PA01-Gm^R^ (wt) or Δ*yfiR*. After 18 hours incubation at 37°C with shaking, dilutions were plated onto LB agar plates containing Congo Red. Colony-forming units were counted following overnight incubation at 37°C. Statistically significant differences between samples were determined using the Mann-Whitney test. Five replicates were run for each sample.

## Results

### The *yfiBNR* operon is an SCV-related locus in *Pseudomonas aeruginosa*

**Figure 1:**
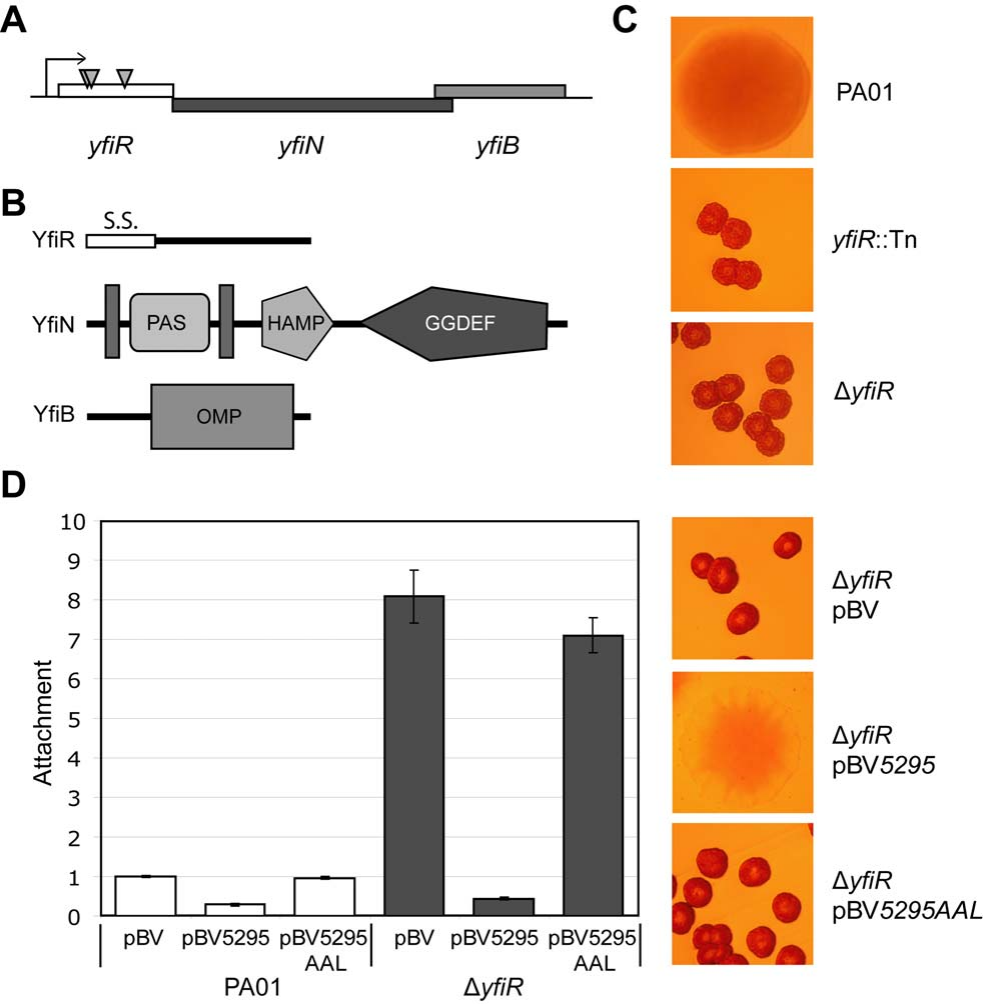
The YfiBNR system of *Pseudomonas aeruginosa.* A) Organization of the *yfiBNR* operon. Transposon insertions in *yfiR* inducing the SCV phenotype are marked with grey triangles. B) Domain organization of the Yfi proteins. S.S. (YfiR) denotes an export signal sequence, vertical grey bars (YfiN) represent transmembrane helices, and OMP (YfiB) denotes the OmpA/Pal-like protein fold. The HAMP and GGDEF domains of YfiN are labeled. C) Colony morphology of the *yfiR*::Tn mutants grown on LB Congo-red agar. The morphology of the non-polar *yfiR* deletion is indistinguishable from the *yfiR*::Tn strains. D) Over-expression of the phosphodiesterase PA5295 abolishes surface attachment and SCV morphology of the Δ*yfiR* mutant. PA5295-AAL denotes an active site mutant of PA5295. Attachment levels are expressed relative to PA01. The graph shows a representative of two independent experiments.

To begin our analysis of the SCV phenotype, we sought to identify those loci in *P. aeruginosa* PA01 whose disruption led to a characteristic SCV morphology and behavior. A comprehensive transposon mutagenesis screen was conducted, selecting for mutants that stably maintained an SCV phenotype on LB Congo Red agar plates (i.e. that could be maintained by serial re-streaking without losing the SCV phenotype). Three independent transposon insertions were mapped to *yfiR* (*PA1121*), the first gene of the predicted three-gene operon *yfiBNR* (Figure 1A). *In silico* analysis predicted that *yfiN* encodes a 47.5 kDa protein with two transmembrane helices flanking a periplasmic PAS-like domain, a HAMP domain and a GGDEF domain with a conserved GGDEF active-site motif. YfiB was predicted to be an 18.4 kDa outer-membrane lipoprotein with a conserved OmpA peptidoglycan binding domain [69], while *yfiR* encodes a 20.7 kDa protein with a probable signal peptide but no predicted tertiary structure [70]–[72](Figure 1B).

Given the established link between c-di-GMP production and SCV and the fact that YfiN has been suggested to function as a DGC [29], we hypothesized that the SCV phenotype of the *yfiR* transposon insertions was caused by up-regulation of YfiN activity. This would occur either via the release of repression by YfiR or as a consequence of polar effects from the Tn insertions on the *yfiN* and/or *yfiB* genes. The *yfiR*::Tn phenotype was successfully complemented *in trans* with a plasmid-borne wild type copy of *yfiR* (data not shown), and an in-frame deletion of *yfiR* yielded an SCV phenotype indistinguishable from that of the transposon mutants (Figure 1C). The *yfiR* transposon mutants (data not shown) as well as the Δ*yfiR* mutant (Figure 1D) showed an almost 10-fold increased propensity for surface attachment. Over-expression of PA5295, a PDE from *P. aeruginosa* [73], but not of an active site mutant (PA5295_AAL_), reduced Δ*yfiR* attachment to wild type levels and abolished the SCV phenotype (Figure 1D). Swimming, swarming, and twitching motility were severely impaired in Δ*yfiR* compared to wild type PA01 (Figure S1). Similarly to the SCV phenotype, these motility defects were fully complemented by expression of *yfiR in cis* or *in trans* (Figure S1D).

**Figure 2:**
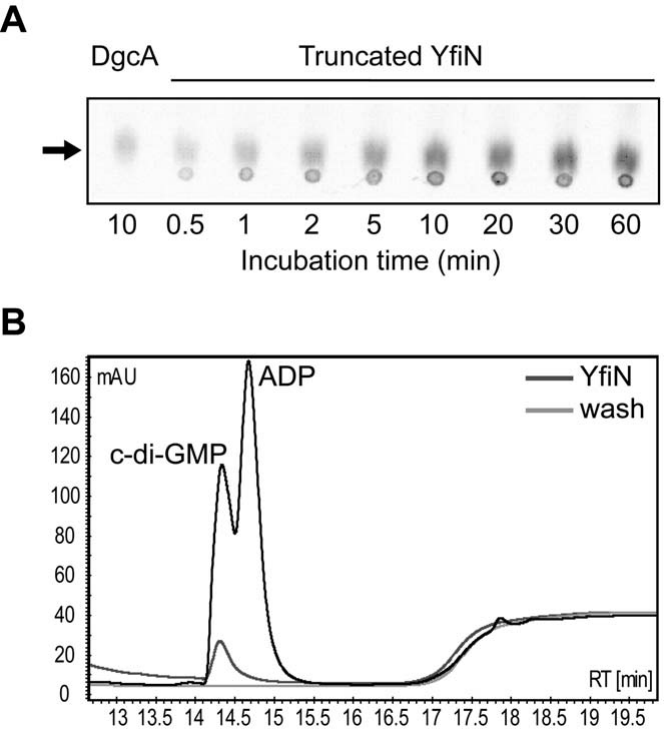
YfiN is a diguanylate cyclase. A) A truncated YfiN lacking the transmembrane and periplasmic parts has DGC activity *in vitro*. C-di-GMP production was observed on TLC plates over time against a positive control (DgcA). The c-di-GMP peak is marked with an arrow. B) YfiN binding of c-di-GMP. Elution and wash fractions from YfiN purification were run on an HPLC column and the absorbance at 254 nm plotted. The elution fraction contains a large c-di-GMP peak, presumably bound by YfiN, and not seen in the wash fraction.

To determine the nature of the YfiBNR system output, a His_6_-tagged version of YfiN lacking the 182 residues corresponding to the transmembrane region (truncated-YfiN-His_6_) was purified and tested for DGC activity. DGC activity was previously suggested for YfiN of *P. aeruginosa* strain PA14 based on HPLC analysis of over-expression strains [29]. In accordance with these findings, truncated-YfiN-His_6_ generated c-di-GMP from GTP, confirming that YfiN functions as a DGC (Figure 2A). HPLC analysis of the effluent co-eluting with the purified truncated-YfiN-His_6_ fraction indicated the presence of large amounts of c-di-GMP (Figure 2B), consistent with the idea that the YfiN GGDEF domain contains a conserved high-affinity binding site utilized for allosteric product inhibition (I-site) [21]. These results indicated that the Δ*yfiR* SCV phenotype is a consequence of derepressed YfiN DGC activity. To confirm this, we determined the cellular concentration of c-di-GMP in wild type and mutant strains. In growing cells of PA01 wild type and a strain lacking YfiN (Δ*yfiBNR*, see below) the concentration of c-di-GMP was 3.54±0.67 pmol/mg protein and 2.39±0.33 pmol/mg, respectively. In contrast, a strain lacking YfiR (289.30±36.53 pmol/mg) or over-expressing *yfiN* from a plasmid (1835.70±235.93 pmol/mg) showed a marked increase of c-di-GMP. Together, these data strongly suggest that YfiR functions to negatively regulate YfiN, and that the Δ*yfiR* SCV phenotype results from de-repression of YfiN DGC activity, leading to increased levels of c-di-GMP.

### YfiB and YfiR inversely control YfiN activity

**Figure 3:**
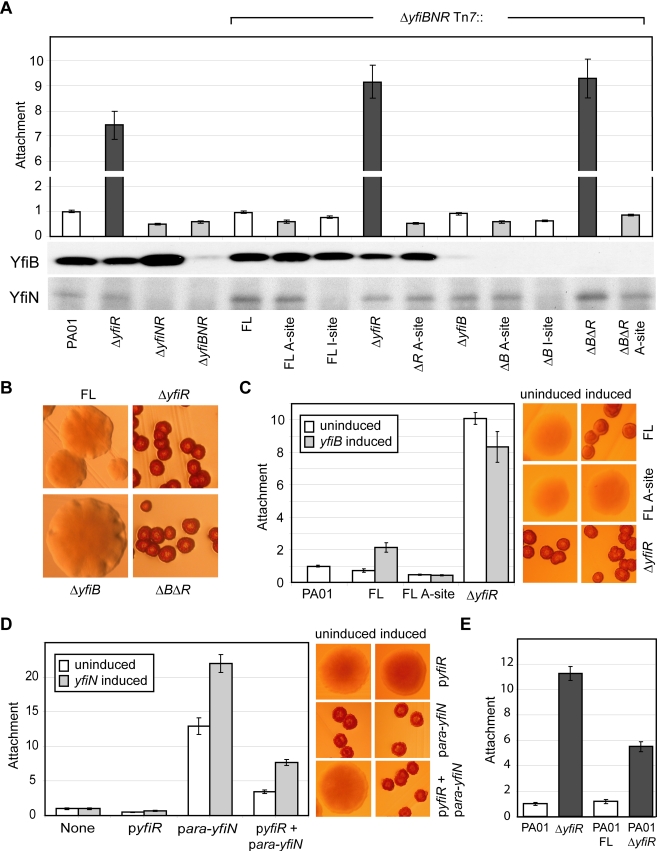
Epistasis and regulation of the YfiBNR system. A) Attachment of *yfiBNR* mutant strains. The bars marked ‘Δ*yfiBNR* Tn*7*::’ depict Tn*7* complementation strains containing variants of the *yfiBNR* operon inserted into the *att-*Tn*7* site of Δ*yfiBNR*. For these, ‘FL’ refers to the full-length *yfiBNR* operon, ‘A-site’ refers to the *yfiN* active-site mutant D330A, and ‘I site’ to the *yfiN* feedback-inhibition-site mutant R319A. Strains without an active copy of *yfiN* (light grey) display reduced attachment compared to those containing active copies of both *yfiN* and *yfiR* (white). Strains missing *yfiR* but containing *yfiN* (dark grey) showed a large increase in attachment. Immunoblots show the levels of YfiB and YfiN present in each strain. B) Colony morphology of selected Tn*7* complementation strains. Deletion of *yfiR* induces a small colony variant phenotype. Deletion of *yfiB* has no effect on morphology, either alone or combined with an *yfiR* deletion. C) Over-expression of *yfiB in trans* induces SCV colony morphology and stimulates PA01 attachment in an *yfiN-* and *yfiR-*dependent manner. *yfiB* expression is induced in Tn*7* complementation strains, which are labeled as in 3A. The colony morphologies of these strains with and without induction of *yfiB* expression are shown on the right. D) YfiR and YfiN expressed *in trans* act antagonistically on PA01 colony morphology and attachment. The X-axis of the graph shows the plasmids present in each case. Colony morphologies with and without induction of *yfiN* expression are shown on the right. E) The full-length *yfiBNR* operon (FL) and Δ*yfiR* Tn*7* complementation constructs were inserted into the *att*-Tn*7* site of PA01. Attachment levels for all assays are shown relative to PA01.

In order to understand the regulatory interplay of the YfiB, YfiN and YfiR proteins, a series of epistasis experiments were performed. In addition to the Δ*yfiR* mutant, Δ*yfiNR* and Δ*yfiBNR* deletion strains were constructed. As expected, both strains displayed wild type colony morphology (data not shown) and reduced attachment (Figure 3A), indicating that YfiN contributes to surface attachment of PA01 wild type under the conditions tested, and that YfiN is the effector of the SCV phenotype in the Δ*yfiR* mutant. A series of *yfiBNR* alleles were then constructed and inserted into the att-Tn*7* locus of Δ*yfiBNR* (a strain that harbors a deletion of the entire *yfi* operon). In this way, the effects of all combinations of *yfiB, yfiN,* and *yfiR* mutations could be tested. Replacement of the full *yfiBNR* operon at att-Tn*7* yielded a phenotype indistinguishable from wild type PA01 (Figure 3A, B). Likewise, complementation with *yfiBN*Δ*R* produced an SCV phenotype with an attachment level comparable to the Δ*yfiR* mutant (Figure 3A, B). These results demonstrated that the Tn*7*-complementation strains can be used to model the effects of *yfiBNR* disruptions *in vivo*.

Comparison of the morphology and attachment of the Tn*7*-based complementation strains led to several observations (Figure 3A, B). Firstly, deletion of *yfiN*, or expression of an *yfiN* active site (A-site) mutant (*yfiN_D330A_*) produced a wild type colony morphology and an attachment level around 60% of wild type, independent of the presence or absence of *yfiR* or *yfiB*. Secondly, deletion of *yfiR* produced the characteristic SCV morphology in any strain with a wild type version of *yfiN*. Deletion of *yfiB* did not affect attachment or colony morphology, and a Δ*yfiRB* double mutant behaved similarly to a Δ*yfiR* single mutant, suggesting that *yfiR* is epistatic over *yfiB* and that YfiB may function upstream of YfiR. Over-expression of *yfiB in trans* led to a two-fold increase in Δ*yfiBNR* Tn*7*::*yfiBNR* attachment (Figure 3C, ‘FL’). This effect was dependent upon the presence of YfiR, and on a functional copy of YfiN; no attachment increase was seen upon *yfiB* expression in either strain Δ*yfiBNR* Tn*7*::*yfiBN*Δ*R* or in strain Δ*yfiBNR* Tn*7*::*yfiBN_D330A_R* (Figure 3C, ‘Δ*yfiR*’ and ‘FL A-site’). These data support the hypothesis that YfiB functions upstream of YfiR. Thirdly, the stoichiometry of YfiR and YfiN appears to be critical for the tight control of the Yfi system (Figure 3D, E). Basal level expression of *yfiN* from the plasmid p-*ara-yfiN* lead to increased levels of YfiN relative to YfiR, and to a SCV phenotype in PA01 wild type. This SCV morphology was suppressed by the introduction of a plasmid expressing *yfiR* (p-*yfiR*). When *yfiN* expression in this strain was induced with arabinose, levels of YfiN once again dominated, leading to an SCV phenotype (Figure 3D). Similarly, adding a second copy of *yfiN* to the PA01 wild type chromosome (inserted into the att-Tn*7* locus) produced an SCV phenotype with a lower level of attachment than Δ*yfiR*, accounting for the chromosomal copy of *yfiR* present, while the wild type phenotype was maintained when additional copies of both *yfiN* and *yfiR* were added (Figure 3E). Finally, an *yfiN* I-site mutation (*yfiN_R319A_*) only partially complemented wild type *yfiN*. Because immunoblot analysis showed reduced levels of the YfiN I-site mutant as compared to wild type (Figure 3A), this may be due to reduced stability of the mutant protein. Despite the reduced effectiveness of *yfiN_R319A_*, we were unable to introduce this allele into a strain that lacked *yfiR*, suggesting that loss of both YfiR-mediated inhibition of YfiN activity and YfiN feedback control produces a highly toxic situation, similar to that observed for feedback negative mutants of *C. crescentus* DgcA [21]. This in turn implies that YfiN, in addition to its regulation by YfiR, is subject to tight feedback control via I-site binding of c-di-GMP. Together, these data strongly indicate that the diguanylate cyclase YfiN is subject to tight negative control by YfiR and that YfiB, directly or indirectly, counteracts this inhibitory effect.

### YfiR is a periplasmic protein

**Figure 4:**
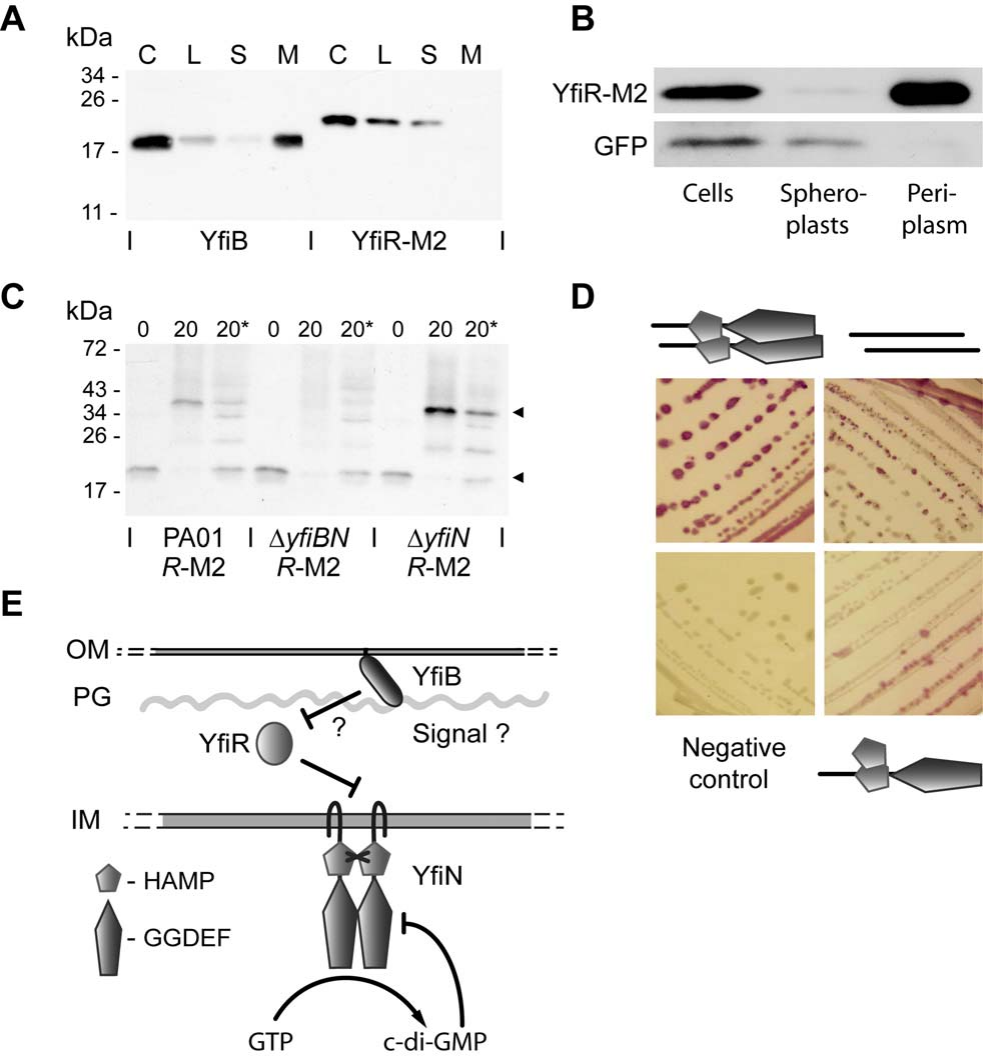
The YfiBNR complex. A) Membrane localization of YfiB and YfiR. Membrane fractionation was carried out with PA01 *yfiR*-M2. The separate fractions are labeled as follows: C  =  whole cell sample, L  =  cell lysate, S  =  soluble fraction, M  =  membrane fraction. YfiB localizes to the membrane fraction, YfiR-M2 to the soluble fraction. B) Periplasmic localization of YfiR. Periplasmic fractionation was carried out with PA01 *yfiR*-M2 pAD6Ω, and YfiR-M2 and GFP were detected by immunoblot analysis. GFP localizes to the spheroplast (cytosolic/membrane) fraction, while YfiR-M2 localizes to the periplasmic fraction. C) *In-vivo* crosslinking of YfiR-M2. Whole cell samples of PA01 *yfiR*-M2, Δ*yfiN yfiR*-M2, and Δ*yfiBN yfiR*-M2 mutant strains were crosslinked by addition of formaldehyde and YfiR-M2 detected by immunoblot analysis. 0  =  sample before crosslinking; 20 =  sample taken 20 min after formaldehyde addition; 20* = 20 min sample, boiled to break crosslinks. Black arrows indicate major bands corresponding in size to an YfiR-M2 monomer (20 kDa) and an YfiR-M2 oligomer (40 kDa), respectively. D) Bacterial two-hybrid analysis of YfiN and YfiR interactions. Positive interactions produce a red color on MacConkey indicator plates. Clockwise from top left, the cartoons denote YfiN-YfiN, YfiR-YfiR and HAMP domain-YfiN interactions. E) A model for YfiBNR function. YfiN is a membrane-localized DGC and is subject to product inhibition and control by YfiR. YfiB activates YfiN, possibly by releasing YfiR-mediated repression. OM and IM refer to the outer and inner membranes, respectively and PG refers to the peptidoglycan layer.

Epistasis experiments indicated that YfiR plays an important role in regulating YfiN activity, possibly by relaying information from YfiB in the outer membrane to YfiN in the cytoplasmic membrane. Such a mechanism would predict that YfiR is located in the periplasm. To test this and due to the absence of an effective YfiR antibody, *yfiR* triple-M2 tagged (*yfiR-M2*) reporter strains were constructed. Insertion of the *yfiR-M2* allele into the att-Tn*7* locus of a Δ*yfiR* mutant restored the wild type phenotype, indicating that YfiR-M2 is fully functional (data not shown). The subcellular location of YfiR-M2 was subsequently investigated upon fractionation of cell extracts by ultracentrifugation followed by immunoblot analysis (Figure 4A). Because YfiB is predicted to localize to the outer membrane [70],[72], it was included as a control. As expected, YfiB almost exclusively localized to the insoluble, membrane-associated fraction. YfiR-M2 was found exclusively in the soluble fraction (Figure 4A). Next, a strain expressing both *yfiR-M2* and *gfp* (PA01 *yfiR-M2* pAD6-Ω) was subjected to periplasmic extraction by osmotic shock, and the fractions were analyzed by immunoblotting. As expected for a cytosolic protein, GFP was detected in the whole-cell and spheroplast fractions only, confirming that cell fractionation did not result in spheroplast lysis. In contrast, YfiR-M2 was found exclusively in the periplasmic fraction (Figure 4B).

To provide further evidence for the periplasmic location of YfiR, an *yfiR-phoA* fusion was analyzed. This construct was able to complement the SCV morphology of a Δ*yfiR* mutant (Figure S2B), indicating that the YfiR section of the fusion protein was fully functional. PA01 wild type containing the full length YfiR-PhoA fusion displayed strong phosphatase activity, with a large proportion of this activity localized to the periplasmic fraction (Figure S2A). In contrast, an *yfiR-phoA* allele lacking the presumable YfiR export signal was unable to complement the Δ*yfiR* SCV morphology and showed little or no phosphatase activity (Figure S2A, B). Finally, an *yfiR-Mcherry* fusion was constructed and shown to fully complement the Δ*yfiR* SCV phenotype (data not shown). When protein localization was visualized by fluorescence microscopy in PA01, the red fluorescent YfiR fusion protein was found exclusively in the cell perimeter (Figure S2C). Together, these data strongly suggest that YfiR is indeed located primarily in the periplasm.

The genetic interaction between YfiB and YfiR together with their presumable location in the outer membrane and periplasm, respectively, prompted us to gather more direct evidence for a link between YfiB and YfiR function. *In vivo* cross-linking experiments were performed, in which YfiR-M2 containing strains were treated with formaldehyde followed by immunoblot analysis of the cross-linked lysates (Figure 4C). In a wild type background, formaldehyde treated samples showed a clear shift of YfiR-M2 from the monomeric state (19.8 kDa, without the signal sequence) to a multimer of approximately 40–42 kDa. This multimer was absent in a strain that lacked both YfiB and YfiN (Δ*yfiBN yfiR-M2*), but was present in a strain that only lacked YfiN (Δ*yfiN yfiR-M2*) (Figure 4C), indicating that YfiB, but not YfiN, is required for YfiR oligomerisation. Immunoblotting with YfiB antiserum failed to detect a band of similar size (data not shown). Whilst the nature of the YfiR multimer is currently unknown, its size is consistent with an YfiR-YfiR homodimer.

Bacterial-two hybrid analysis failed to provide evidence for direct interaction between YfiR and the isolated periplasmic or cytoplasmic domains of YfiN, and between YfiB and any other protein variant (Table S3). Given the different subcellular localization of these proteins some of these negative results might reflect limitations in the experimental procedure. A truncated variant of YfiR missing the predicted signal peptide (residues 1-34) [70] interacted strongly with itself (Figure 4D), consistent with the results obtained from cross-linking experiments (see above). Also, while no interaction was observed with the full-length YfiN (data not shown), a truncated version of YfiN missing its transmembrane region (residues 1-182) formed strong interactions with itself (Figure 4D), arguing that, like other DGCs [74]-[76], YfiN functions as a dimer and that the membrane spanning part of YfiN negatively controls YfiN dimerisation. The isolated HAMP domain, but not the isolated GGDEF domain of YfiN also interacted with the truncated YfiN variant, suggesting that the HAMP domain is involved in YfiN multimerization (Figure 4D). Together, these data suggest that YfiB and YfiR are localized outside of the cell and together control YfiN activity.

### The YfiBNR system controls the Pel and Psl exopolysaccharide systems

**Figure 5:**
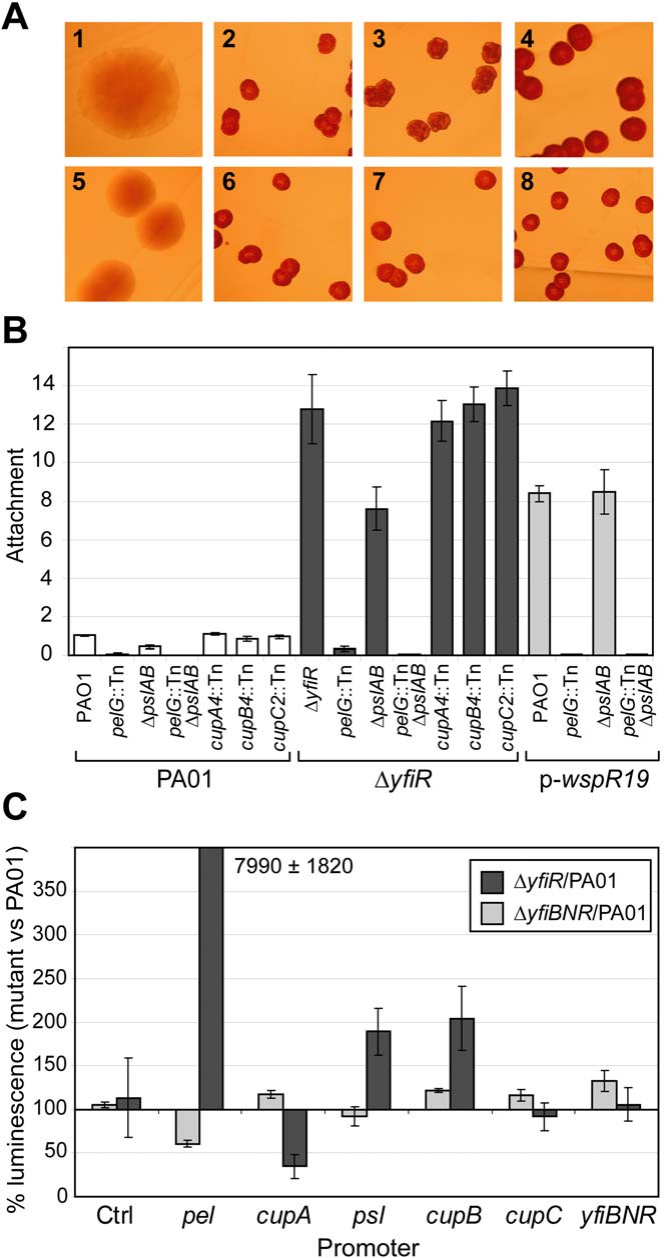
Downstream components of the YfiBNR system. A) Colony morphologies of mutants lacking potential downstream targets of the YfiBNR system grown on LB Congo Red agar. 1 =  PA01, 2 =  Δ*yfiR*, 3 =  Δ*yfiR pelG*::Tn, 4 =  Δ*yfiR* Δ*pslAB*, 5 =  Δ*yfiR* Δ*pslAB pelG*::Tn, 6 =  Δ*yfiR cupA4*::Tn, 7 =  Δ*yfiR cupB4*::Tn, 8 =  Δ*yfiR cupC2*::Tn. B) Attachment of mutants lacking potential downstream targets of the YfiBNR system, relative to PA01. C) YfiBNR effects on downstream gene transcription. Values are expressed as the percentage of luminescence reporter gene expression in Δ*yfiR* or Δ*yfiBNR*, as compared to PA01 wild type. The control strain contains pME6032-*luxCDABE*. Transcription of *pel* is massively up-regulated in Δ*yfiR*, and down regulated in Δ*yfiBNR*.

Elevated levels of c-di-GMP have been linked to a number of biofilm-promoting systems including Pel and Psl exopolysaccharides [12] and the Cup fimbrial adhesins [9],[11],[26]. To identify potential downstream targets of YfiN that contribute to the SCV behavior, the Δ*yfiR* deletion allele was combined with disruptions in several of these c-di-GMP output systems. Disruption of *pel* (*pelG*::Tn) or *psl* (Δ*pslAB*) strongly attenuated the SCV phenotype of a Δ*yfiR* mutant, resulting in strains with altered colony morphologies and Congo Red binding on LB-agar plates (Figure 5A), as well as significantly reduced attachment (Figure 5B). Importantly, a *pelG::Tn* Δ*pslAB* Δ*yfiR* triple mutant exhibited a smooth colony morphology, a marked loss of Congo Red binding and complete abolishment of surface attachment. In contrast, mutational disruption of the CupA (*cupA4*::Tn), CupB (*cupB4*::Tn), or CupC (*cupC2*::Tn) fimbrial adhesins showed no discernable effects on the Δ*yfiR* SCV phenotype (Figure 5A, B). Over-expression of *wspR19*, which encodes a constitutively active DGC [52] produced high levels of attachment, comparable to the Δ*yfiR* mutant. However, in this case up-regulation of Pel alone seems to be responsible for increased attachment, as disruption of *psl* produced little effect (Figure 5B). Disruption of *pel* and *psl* in the Δ*yfiR* background failed to restore swimming and twitching motility and only partially restored swarming motility (Figure S1). This suggested that the impairment of cellular motility in the Δ*yfiR* mutant is not an indirect consequence of Pel and Psl exopolysaccharide overproduction and that additional, motility-related systems are affected.

To investigate the relationship between YfiBNR and its downstream targets in more detail, gene transcription in different Δ*yfi*-backgrounds was probed using *lux*-promoter fusion constructs (Figure 5C). Compared to PA01, deletion of the entire *yfiBNR* operon led to a 40% drop in *pel* transcription level, which likely contributes to the reduced attachment level seen in Δ*yfiBNR* (Figure 3A). In contrast, in a Δ*yfiR* background, *pel* transcription was massively induced compared to PA01. Transcription of the *psl* and *cupB* operons was subject to a modest two-fold increase in Δ*yfiR* over wild type levels. Interestingly, *cupA* transcription was strongly inhibited in this strain under the conditions tested.

Electron micrographs of PA01 and Δ*yfiR* strains (Figure S3) showed cells embedded in a dense matrix of thin fibres. This matrix was markedly thicker for the Δ*yfiR* mutant, and the cells in this case appeared to form a more structured biofilm. In PA01, disruption of both *pel* and *psl* greatly reduced biofilm formation (Figure S3). In the Δ*yfiR* background, disruption of *pel* did not abolish biofilm formation, but the biofilm that was formed displayed few extracellular fibres, suggesting that Pel is a component of the extracellular matrix. Δ*yfiR* Δ*pslAB* produced both a biofilm and extracellular fibres, but the latter in this case were irregularly distributed and had a ‘ragged’ appearance, in contrast to the thick, even matrix seen with the parental Δ*yfiR* strain (Figure S3). Little or no biofilm formation was observed when both *pel* and *psl* were disrupted in either *yfiR* background (data not shown). Together, these data indicate that de-repression of YfiN leads to the activation of the Pel and Psl exopolysaccharide systems, and that most of the morphological and behavioral characteristics of the Δ*yfiR* SCV are mediated by the up-regulation of Pel and Psl.

### YfiN-dependent up-regulation of Pel and Psl exopolysaccharide systems confers resistance against phagocytosis

The clinical SCV phenotype is associated with persistence in CF lung infections [7]. In addition, SCV is a highly aggregative phenotype associated with over-production of exopolysaccharide in both clinical [4] and lab strains [77]. To determine whether *yfiBNR* mutations give rise to SCVs in CF lung infections, ten SCV strains isolated from the sputum of CF patients were transformed with pMR20-*yfiR-M2*. The SCV phenotype of one of these strains, ClinSCV-110, was abolished by *yfiR-M2* expression, similarly to the Δ*yfiR* SCV mutant (Figure S4). This strongly suggested that the SCV phenotype in this case arose as the consequence of a mutation in the *yfi* operon, and validates the use of the Δ*yfiR* mutant as a model SCV for subsequent *in vivo* analyses.

**Figure 6:**
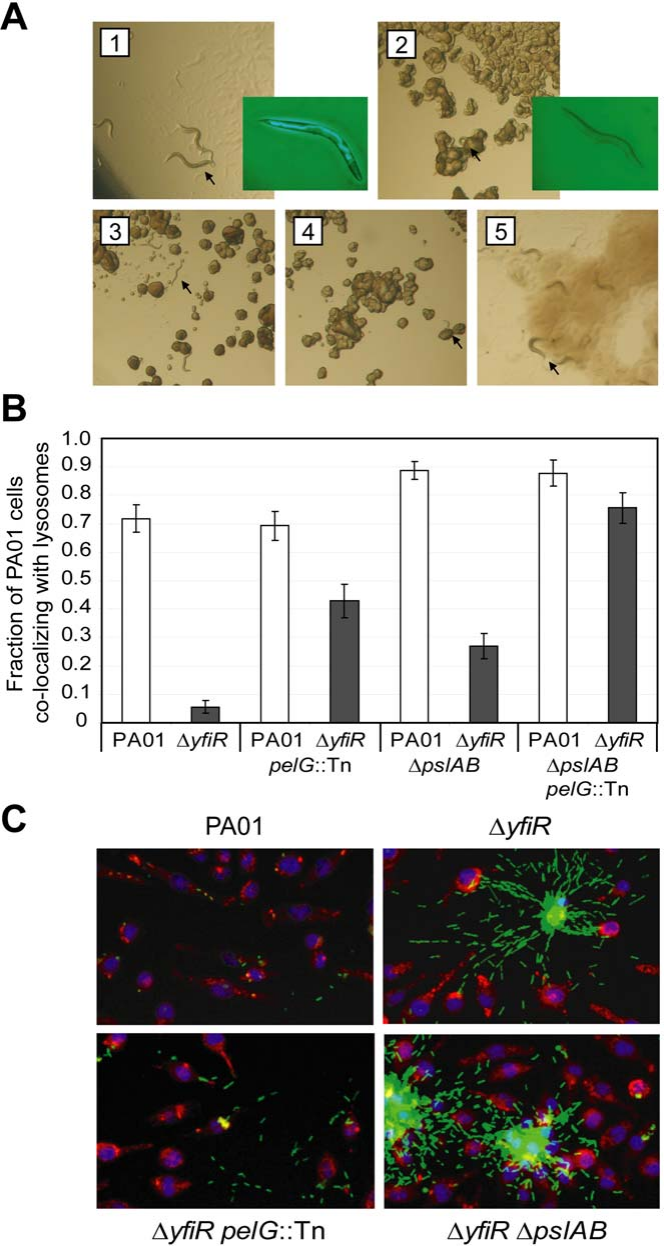
YfiN-mediated SCVs resist nematode predation and phagocytosis. A) SCV resistance to nematode predation. Panel 1 shows *C. elegans* incubated with PA01 as a food source for 72 hours. Panels 2 to 5 show the corresponding experiments with Δ*yfiR* (2), Δ*yfiR pelG*::Tn (3), Δ*yfiR* Δ*pslAB* (4) and Δ*yfiR pelG*::Tn Δ*pslAB* (5), respectively. Eggs and mature adults were seen with PA01 (1) and the double exopolysaccharide mutant (5) only. Worms and larvae are indicated with black arrows. Insets to panels 1 and 2 show nematodes incubated for three hours with GFP-labeled PA01 or Δ*yfiR*, respectively. B) Macrophage absorption assay. Values shown are the fraction of J774 macrophages whose lysosomes co-localize with at least one bacterium. White bars show PA01 background strains, dark grey bars denote a Δ*yfiR* background. C) Surface attachment patterns of Δ*yfiR* exopolysaccharide mutants. Panels show attachment to polystyrene of GFP-labeled bacteria (green) following three hours growth at 37°C. J774 macrophages are stained with DAPI (blue) and Lysotracker (red).

To assay the persistence behavior of the Δ*yfiR* mutant and to test the hypothesis that persistence was related to the exopolysaccharide components of the biofilm, we analyzed the interaction of the Δ*yfiR* mutant with both murine immune cells and external predators. *Caenorhabditis elegans*, incubated for 72 hours on minimal agar plates onto which drops of PA01 had been spotted grew to maturity with no apparent adverse effects (Figure 6A, panel 1). In contrast, nematodes incubated with Δ*yfiR* were starved, with many individuals in the dauer larva stage, a stage reflecting low food resources (Figure 6A, panel 2). When incubated for three hours on GFP-labeled cells, the gut of nematodes fed with PA01 was filled with green bacteria (Figure 6A, panel 1 inset), while nematodes fed on Δ*yfiR* showed almost no bacteria in the gut (Figure 6A, panel 2 inset). Disruption of either the *pel* or *psl* operon alone was insufficient to overcome the resistance of the Δ*yfiR* mutant to effective nematode scavenging (Figure 6A, panels 3-4). However, a Δ*yfiR pelG*::Tn Δ*pslAB* triple mutant was consumed readily; nematodes incubated with this strain grew to maturity in the same way as with wild type PA01 (Figure 6A, panel 5). These results demonstrate that the Δ*yfiR* SCV phenotype confers exopolysaccharide-dependent resistance to ingestion by *C. elegans*.

To investigate the interaction of Δ*yfiR* mutant cells with professional phagocytic cells of the immune system, J774 macrophages were incubated with GFP-labeled PA01 variants. When incubated for three hours with PA01 wild type, 71.9±4.8% of the macrophages had taken up at least one bacterium. In contrast, only 5.6±2.3% of the macrophages had phagocytosed Δ*yfiR* mutant cells, indicating that the Δ*yfiR* SCV phenotype confers substantial resistance to macrophage phagocytosis (Figure 6B). To investigate the contribution of the Pel and Psl systems to interference with internalization, the assay was repeated with Δ*yfiR pelG::Tn* and Δ*yfiR* Δ*pslAB* mutant strains. The percentage of macrophages that had taken up at least one bacterium rose to 42.8±6.0% for the Δ*yfiR pelG::Tn*, 27.0±4.5% for the Δ*yfiR* Δ*pslAB*, and to 75.6±5.3% for the Δ*yfiR* Δ*pslAB pelG::Tn* triple mutant (Figure 6B). The observation that phagocytosis of the Δ*yfiR* SCV mutant was fully restored upon disruption of the *pel* and *psl* loci not only provided further evidence that these exopolysaccharides represent one of the main cellular outputs for the YfiBNR system, but in agreement with previous studies [78],[79] also confirms the notion that matrix engulfment enables bacterial cells to escape the host immune response.

In addition to interfering with internalization, the different *yfi*, *psl*, and *pel* mutants displayed markedly different surface attachment phenotypes in this assay. Clusters of extracellular Δ*yfiR* (*pel* ^+^ *psl* ^+^) mutant cells observed in the macrophage phagocytosis assay showed a specific ‘spider-like’ organization, where cells spread out from the central region of aggregation along a number of defined trajectories (Figure 6C). The pattern for the Δ*yfiR* Δ*pslAB* mutant was markedly different in that cells randomly spread out from the central region in every direction, lacking the radial tracks seen for Δ*yfiR* spreading. In contrast, the Δ*yfiR pelG::Tn* mutant, although unable to form large clusters of aggregated cells, appeared to be organized into similar linear trajectories as the Δ*yfiR* SCV mutant (Figure 6C). No particular cell arrangement was observed for the Δ*yfiR* Δ*pslAB pelG::Tn* triple mutant, although few non-phagocytosed cells were seen for this strain (Figure 6B). These observations suggest that Pel and Psl not only contribute to cell adherence, biofilm formation, and protection against phagocytosis, but also mediate a specific architecture of surface attached microcolonies.

Given the clear differences in physical interaction between macrophages and SCVs, we sought to test whether these differences were mirrored in the internal response of the immune cells. To test whether the Δ*yfiR* SCV phenotype affected macrophage activation or cytotoxicity, nuclear NF-κB translocation (Figure S5) and LDH (lactate dehydrogenase) release (Figure S6) was determined in J774 macrophages infected with PA01 wild type and mutant cells, respectively. No significant difference in macrophage activation was seen between samples infected with PA01 or the Δ*yfiR* mutant. When siderophore production was measured for the Δ*yfiR* strain, increased levels of pyoverdin and pyochelin were found compared with wild type PA01 (Figure S7). An increase in the production of siderophores and other excreted ‘scavenger’ molecules would appear to be consistent with the persistence phenotype proposed for SCV strains. The Δ*yfiR* strain also showed increased production of the phenazine pyocyanin (Figure S7).

### The Δ*yfiR* SCV phenotype persists in a murine subcutaneous catheter infection

**Figure 7:**
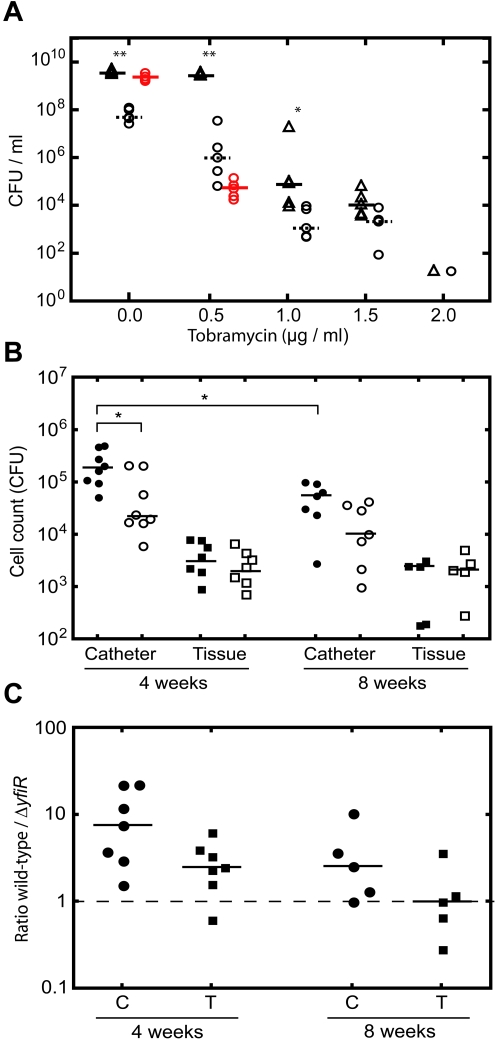
YfiN-mediated SCVs contribute to persistence *in vitro* and *in vivo.* A) Tobramycin survival assay with PA01 wild type (open triangles) and Δ*yfiR* mutant (open circles). Δ*yfiR* suppressor colonies are shown as red circles. Statistically significant differences between PA01and Δ*yfiR* are marked with asterisks (** = p<0.01, * = p<0.05). B) Single infections with PA01 wild type (black symbols) and Δ*yfiR* mutant (open symbols). Catheter samples (circles) are presented as cfu/ml, tissue samples (squares) as cfu/mg tissue. Statistically significant differences are marked with an asterisk (* = p<0.05). C) Competition experiments with PA01 wild type and Δ*yfiR* mutant. The graph shows the ratio of wild type to Δ*yfiR* mutant colonies recovered from catheters (C, circles) and tissue (T, squares) after four and eight weeks, respectively. Values above 1 indicate greater numbers of wild type colonies, values below 1 greater numbers of SCVs.

Although the auto-aggregative and slow growing SCV morphotypes are at a considerable disadvantage under conditions that permit rapid growth, they are able to persist *in vivo* [7]. This phenomenon might be explained by conditions in the host environment (e.g. immune system attack or antimicrobial chemotherapy) that put an even higher burden on rapidly growing, non-adherent strains and thus provide selection for SCVs. To test this, we compared the competitive behavior of PA01 wild type and Δ*yfiR* SCV strains *in vitro* and *in vivo*. When grown in LB the Δ*yfiR* SCV strain was significantly outperformed by the wild type. Also, suppressors with wild type colony morphology quickly arose in every Δ*yfiR* sample, forming the majority of the cell population after 18 hours incubation (Figure 7A). The addition of increasing concentrations of tobramycin in the sub-inhibitory range led to a reduction of both wild type and Δ*yfiR* cell numbers. However, wild type growth decreased at a much steeper rate and at 1.5 µg/ml tobramycin, no significant difference in cfu numbers was observed between the two strains (Figure 7A). Likewise, in the presence of tobramycin the number of suppressors arising from Δ*yfiR* dropped sharply with no suppressors arising at concentrations above 0.5 µg/ml of the inhibitor. Thus, the fitness disadvantage of the Δ*yfiR* SCV is strongly reduced in the presence of sub-inhibitory concentrations of tobramycin. Two observations indicate that this effect is not linked to some form of antibiotic tolerance of the Δ*yfiR* SCV, but rather reflects converging fitness during slow growth under stressful conditions. Firstly, a similar relative increase of Δ*yfiR* fitness compared to wild type was observed when cells were grown at reduced temperatures (data not shown). Secondly, no differences in MIC were seen between PA01 and Δ*yfiR* for tobramycin or for any of the other antibiotics tested (Figure S8).

Next, we analyzed the Δ*yfiR* mutant in a mouse catheter model with respect to its *in vivo* persistence behavior. Total bacterial numbers and colony morphologies were scored in the catheters and in the surrounding tissue four and eight weeks after infection with PA01 wild type, Δ*yfiR* and Δ*yfiBNR* mutants, either individually or in competition. When strains were infected individually, total numbers of Δ*yfiR* mutants scored within the catheter were significantly lower after four weeks as compared to wild type (Figure 7B). In contrast, no significant difference was observed between the corresponding tissue samples (Figure 7B). Likewise, in four week competition experiments, the Δ*yfiR* mutant was strongly outperformed by wild type in the catheter but less so in the surrounding tissue (Figure 7C). After eight weeks of infection, viable counts of wild type cells were significantly reduced in the catheter compared with four-week infections. However, no significant drop in cfu/ml was noted for the Δ*yfiR* mutant between four and eight weeks, accounting for an improved relative performance of the SCV mutant (Figure 7B). In competition assays after eight weeks the wild type/Δ*yfiR* ratio decreased relative to the four-week results in both catheter and tissue samples. While a small (non-significant) disadvantage was still seen for the Δ*yfiR* mutant in the catheter, no such disadvantage was noted for the eight-week tissue samples (Figure 7C). The improved relative performance of the Δ*yfiR* mutant appeared to be due to a reduction in the number of wild type cfus, rather than an increase in SCVs. Importantly, *in vivo* no suppressors of the SCV morphology arose for any of the Δ*yfiR* mutant samples over the entire course of the experiment, in contrast to the rapid emergence of suppressors *in vitro* (Figure 7A). No significant differences were seen between wild type and the Δ*yfiBNR* strain after four weeks, either in single infections or competition experiments (data not shown).

Together this demonstrates that the slow-growing Δ*yfiR* SCV mutant, although suffering from a tremendous growth disadvantage, shows characteristic persistence behavior under antibiotic selection and during prolonged infection *in vivo*.

## Discussion

In this work we identify and characterize YfiBNR, an important regulatory system involved in *Pseudomonas aeruginosa* biofilm formation and *in vivo* persistence. Our research suggests that YfiB and YfiR are upstream regulatory components of the YfiN diguanylate cyclase, which represents the cellular readout of the system. Activation of YfiN results in c-di-GMP production, and the activation of several downstream targets affecting cell motility and exopolysaccharide production. We identify YfiR as a key player in containing YfiN activity. Mutants lacking YfiR strongly activate YfiN leading to a characteristic SCV phenotype distinguished by slow growth, reduced motility, a highly aggregative and wrinkled colony morphology, strong attachment to surfaces, and persistence *in vivo*. Epistatic and biochemical analyses led to a model for YfiBNR function and control (Figure 4E), wherein YfiR acts as small periplasmic protein that is able to tightly repress the activity of the membrane-localized DGC YfiN. YfiB counteracts YfiR-mediated repression of YfiN and hence leads to increased c-di-GMP production. YfiB is a homolog of the Pal family of lipoproteins that are anchored either in the inner or outer membrane and directly interact with the peptidoglycan structure via a conserved peptidoglycan-binding site [69]. Because mutations of the *tol-pal* genes induce hypersensitivity to external stress-factors [80], it is possible that YfiB plays a role in sensing envelope stress, and, in response, stimulates an SCV response by relaying changes in the outer cell layer to YfiN via YfiR. The observation that DGCs like YfiN are active as dimers [74],[75], together with the possible YfiB-dependent dimerisation of YfiR is consistent with the idea that YfiR represses YfiN activity by interfering with its oligomerisation state. In this model, YfiB activates YfiN by promoting the oligomerisation of YfiR, freeing YfiN to form active dimers and thus produce c-di-GMP. An interesting alternative regulatory mechanism for YfiN has recently been proposed [81]. In this scheme, YfiN activity is controlled by de-phosphorylation of the periplasmic domain by the tyrosine phosphatase TpbA. This mechanism could in principle work in parallel with YfiR/YfiB-mediated control of YfiN activity, or as part of the same regulatory pathway. Further experiments are required to fully address the modes of action of YfiB and YfiR, and the regulatory hierarchy of the Yfi system.

While the signals that activate the Yfi system remain to be identified, several of the downstream targets are well known. Genetic experiments indicated that YfiN-mediated induction of the Pel and Psl exopolysaccharides plays a central role in the SCV morphotype. Disruption of either exopolysaccharide operon led to a partial phenotype, while disruption of both systems produced colonies with wild type morphology. Transcription of *pel* was massively increased in the Δ*yfiR* strain compared to wild type; much of the increase in Pel exopolysaccharide production in Δ*yfiR* may be as a consequence of this increased transcription. Despite the substantial contribution of Psl to the Δ*yfiR* phenotype, *psl* transcription was only modestly increased in this mutant, suggesting that c-di-GMP mediated Psl stimulation in Δ*yfiR* may be at a different regulatory level. In an *yfiBNR* deletion strain, *pel* transcription and surface attachment drop by approximately 40% when compared to wild type, arguing that the Yfi system contributes significantly to Pel and Psl-dependent biofilm formation under standard laboratory conditions. In contrast to *pel* and *psl* transcription, *cupA* transcription was strongly repressed in the Δ*yfiR* mutant under the conditions tested. Similar reciprocal relationships between exopolysaccharide and fimbrial gene transcription have been noted previously in *P. aeruginosa* [82] and *E. coli* [83]. The physiological relevance of this is unknown, although one may speculate that *cupA* repression is a compensatory response to the enhanced production of Pel and Psl in the SCV state. The patterns of biofilm formation and surface attachment observed suggested critical roles for Pel and Psl in the formation and organization of SCV colonies, respectively. The Δ*yfiR* (*pel* ^+^ *psl* ^+^) mutant strain formed clusters of aggregated cells, from which cells radiated along defined trajectories. Whereas disruption of the *pel* operon appeared to primarily affect cell-cell association, a *psl* mutation did not affect the number or size of cell clusters, but rather their organization, with cells spreading out from the central region in an apparently random fashion. While Pel appears to provide the principal structural element of the *P. aeruginosa* SCV biofilm, Psl may function more as a scaffold to mediate a specific biofilm architecture and to ensure organized and effective biofilm construction, in agreement with previous studies [84].

The control of Pel exopolysaccharide biosynthesis appears to be complex, as several c-di-GMP signaling components have been implicated with this process [12],[13],[25],[32],[36],[37],[40],[42],[45]. Recently, Starkey and co-workers [13] described two classes of lab-derived SCVs, which in many respects resemble the phenotype of the Δ*yfiR* mutant. While the molecular nature of class B SCVs is unclear, members of class A could be complemented by *wspF*, arguing that they were caused by mutations that activate the WspR DGC pathway [12]. Both classes of SCVs over-produce exopolysaccharides and display a transcriptional profile for *pel* and *psl* similar to that observed here for the Δ*yfiR* SCV. Likewise, CupA fimbrial adhesins do not play a role in the SCV phenotype of either sub-class [13]. Loss of function mutations in *yfiR* are thus prime candidates for class B SCVs. Clinical and lab-derived SCVs are physiologically similar, with a number of characteristic phenotypes in common [4],[13]. Because of the relatively high frequency of loss of function mutations, both *wspF* and *yfiR* might be important targets for genetic adaptations leading to clinical SCV development. In support of this, *wspF* mutations have already been identified in long-term *P. aeruginosa* CF isolates [2]. Similarly, the SCV phenotype of strain ClinSCV-110 appears to derive from an *yfi* mutation. In addition to ClinSCV-110, another potential candidate for a clinical *yfiR* SCV has also been isolated, although the genetic basis for this SCV has not yet been identified [11]. The clinical isolate SCV-20265 reverted to a wild type phenotype upon mutation of *yfiN*, strongly implicating the Yfi system in the generation of the SCV phenotype. Surprisingly, CupA adhesin is an important component of the SCV phenotype of SCV-20265, contrary to our findings for the Δ*yfiR* mutant [11]. A possible explanation for this is that the downstream targets of YfiN in SCV-20265 may differ from those seen in the laboratory strain.

The SCV phenotype caused by de-repression of the YfiN DGC conferred a marked resistance to nematode scavenging and phagocytosis by macrophages. This phenotype was shown to be strongly dependent on exopolysaccharide production, with concomitant disruption of the *pel* and *psl* operons restoring the ability of nematodes to scavenge, and macrophage phagocytosis levels to those of wild type PA01. There is evidence to suggest that this resistance to phagocytosis derives from the protection offered to each individual cell by the exopolysaccharide, rather than being a consequence of cell aggregation. First, macrophages are able to take up particles larger than many of the SCV cell aggregates [85]. Second, in contrast to wild type cells, many single cells of the SCV strain remained un-internalized even after several hours of co-incubation with macrophages (Figure 6C). This phenotype is in agreement with the role of mannose receptors as major phagocytic receptors for *P. aeruginosa* [86]. Exopolysaccharides may sterically hinder binding or alter macrophage surfaces and internalization via mannose receptors. Enhanced resistance to the immune system may help to explain the persistence of SCV infections in the subcutaneous catheter infection model. Although bacterial counts of the SCV strain were initially lower due to the severe growth impediment of this strain, SCV cell numbers remained constant over eight weeks in both catheter and neighboring tissue samples. In contrast, numbers of wild type cells rapidly declined during the same time window. The exact molecular basis for SCV persistence *in vivo* is currently unclear. However, given the resistance to macrophage phagocytosis seen *in vitro*, and in the absence of complicating external factors such as antibiotic stress, it is tempting to speculate that the competitive advantage of SCVs compared with wild type *P. aeruginosa* cells in long term infections is due to immune evasion. The better relative performance of the YfiN-mediated SCV strain in tissue compared with catheters would suggest that exopolysaccharide protection against phagocytosis plays a more important role in the surrounding tissue, consistent with an increased exposure to the vascularized immune system. Cells in a bacterial biofilm have been shown to be highly tolerant of antibiotic treatment [87], with tolerance attributed to characteristics including biofilm structure [88], persister cells [89] and slow growth rates [90]. Resistance to phagocytosis and clearance, especially following aggressive antibiotic therapy when the bacterial load of an infection is lower, and the relative risk of exposure to immune cells is correspondingly higher, might thus contribute to the SCV persistence phenotype in CF patients.

Although the persistence effect of the *ΔyfiR* SCV strain observed in these experiments was significant, even after eight weeks of infection wild type counts were still comparable with those of the mutant. Likewise, no SCVs arose in the animals from wild type samples, contrary to the situation in CF patients [5],[6]. Longer periods of infection, higher infection load, and external challenges (e.g. antibiotic stress) may increase the competitive advantage of SCVs, resulting in a higher proportion of SCVs and the evolution of new SCV morphotypes from the wild type population. In support of this hypothesis, exposure to sub-lethal concentrations of tobramycin was shown to significantly reduce the number of suppressors arising in an *in vitro* culture of the Δ*yfiR* SCV strain, and at higher concentrations, decreased the relative fitness advantage of wild type PA01 over SCV. In addition, it is possible that the comparatively large number of persister cells found in biofilms could further increase the advantage of SCV-generating mutations in the presence of antibiotic stress [89]. Work is ongoing to investigate these possibilities.

In this study, we have identified and characterized a novel c-di-GMP regulatory system, and investigated its role both in wild type *P. aeruginosa* and in the SCV morphotype. While homologous systems have been mentioned previously in the literature [91],[92], this work represents the first comprehensive experimental analysis of the YfiBNR system. The YfiN cyclase is tightly controlled by its upstream regulatory system, while loss of this control leads to the formation of a strong SCV phenotype. In the SCV form, *P. aeruginosa* adopts a hyper-adherent, aggregative lifestyle, with significant implications for immune evasion and ultimately for long term persistence of infection. Further research should determine the extent to which these findings are relevant to chronic colonization in patients with CF and implant-borne infections.

## Supporting Information

Table S1 Strains and plasmids used in this study
(0.11 MB PDF)

Table S2 Primers used in this study
(0.02 MB PDF)

Table S3 Bacterial-two-hybrid results
(0.02 MB PDF)

Figure S1 Motility of the Δ*yfiR* and exopolysaccharide mutant strains. A) Swimming, swarming and twitching motility of Pel and Psl exopolysaccharide mutants in a wild type PA01 background. B) Motility in the Δ*yfiR* mutant background. Motility is abolished in the Δ*yfiR* mutation (5), but swimming and twitching are partially restored by disruption of Pel and Psl production (6-8). C) PA01 motility (1, 9) is unaffected by expression of *yfiR in trans* (10). D) The motility defect of the Δ*yfiR* mutant (5, 11) is restored by expression of *yfiR in cis* (13) or *in trans* (12).
(0.10 MB PDF)

Figure S2 YfiR localizes to the periplasm. A) Alkaline phosphatase activity in whole-cell, spheroplast and periplasmic fractions is shown for PA01 strains expressing *yfiR-phoA* fusions. Activity is seen in all fractions with full-length YfiR-PhoA, but not with a truncated YfiR allele missing the first 33 residues including the signal sequence. An empty vector is used for the control. Values are expressed as A405/OD600/min ± standard error. B) Colony morphology of Δ*yfiR* mutants expressing *yfiR-phoA* fusions. Full-length YfiR-PhoA successfully complements the SCV phenotype of the Δ*yfiR* strain, while truncated YfiR-PhoA does not. C) YfiR-MCherry localizes to the periplasm, as determined by fluorescence microscopy.
(0.04 MB PDF)

Figure S3 Scanning electron micrographs of PA01 and Δ*yfiR* exopolysaccharide mutants. The scale bars in each panel represent 2 µm.
(0.12 MB PDF)

Figure S4 A clinically derived SCV responds to *yfiR* expression. Expression of *yfiR-M2* from pMR20 reverts the autoaggregative, Congo Red binding phenotypes of ClinSCV-110. PA01 and Δ*yfiR* strains are shown for comparison.
(0.03 MB PDF)

Figure S5 NF-κB activation by macrophages incubated with ΔyfiR and wild type PA01. NF-κB activation level in J774 macrophages was unchanged between the Δ*yfiR* mutant and wild type PA01. The control lane shows activation levels for macrophages incubated without bacteria. Values are expressed as the percentage of cells showing NF-κB translocation to the nucleus ± standard error.
(0.01 MB PDF)

Figure S6 Cytotoxicity of the Δ*yfiR* mutant strain. No significant differences in LDH release from J774 macrophages were seen between the Δ*yfiR* SCV mutant and wild type PA01 under the conditions tested. Values are expressed as a percentage of the LDH released from a fully-lysed positive control sample. The graph shows the combined results of three independent experiments ± standard deviation.
(0.01 MB PDF)

Figure S7 Pyocyanin and siderophore production by the Δ*yfiR* strain. Values are shown relative to PA01 wild type, ± standard error.
(0.01 MB PDF)

Figure S8 Antibiotic susceptibility of the Δ*yfiR* mutant strain. Inhibition zones for different antibiotic discs are shown for PA01 and the Δ*yfiR* mutant strains. Values shown are for the diameter of the inhibition zone in mm, and show the mean of three samples ± standard error in each case.
(0.02 MB PDF)
