# Supplementary material for: YfiBNR Mediates Cyclic di-GMP Dependent Small Colony Variant Formation and Persistence in Pseudomonas aeruginosa
Source: PLoS Pathog. 2010 Mar 12;6(3):e1000804. doi: 10.1371/journal.ppat.1000804 (PMC2837407; doi:10.1371/journal.ppat.1000804)
Supplement: Table S1 — Strains and plasmids used in this study (0.11 MB PDF) [file ppat.1000804.s001.pdf]

Table S1: Bacterial strains and plasmids

Strains	Description	Reference
P. aeruginosa		
PA01	Wild-type *P. aeruginosa*	[1]
Δ*yfiR*	PA01 with *yfiR* deleted	this study
Δ*yfiNR*	PA01 with *yfiNR* deleted	this study
Δ*yfiBNR*	PA01 with *yfiBNR* deleted	this study
yfiBNR-complementation strains	Δ*yfiBNR* with *yfiBNR* complementation operons inserted at the Att::Tn7 locus. Ten strains in total	this study
PA01 *yfiR*-M2	Δ*yfiR* with *yfiR*-M2 inserted at the Att::Tn7 locus	this study
Δ*yfiN yfiR*-M2	Δ*yfiNR* with *yfiR*-M2 inserted at the Att::Tn7 locus	this study
Δ*yfiBN yfiR*-M2	Δ*yfiBNR* with *yfiR*-M2 inserted at the Att::Tn7 locus	this study
PA01-Gm^R	Gm^R, PA01 with the gentamycin resistance cassette inserted at the Att::Tn7 locus	this study
PA01 *cupA*::Tn	Tet^R, PA01 with the lacZ-Tn5 transposon inserted into *cupA4*	[2]
PA01 *cupB*::Tn	Tet^R, PA01 with the phoA-Tn5 transposon inserted into *cupB4*	[2]
PA01 *cupC*::Tn	Tet^R, PA01 with the lacZ-Tn5 transposon inserted into *cupC2*	[2]
PA01 *pelG*::Tn	Tet^R, PA01 with the phoA-Tn5 transposon inserted into *pelG*	[2]
PA01 Δ*pslAB*	Gm^R, PA01 containing the WFPA60 Δ*pslAB* cassette	[3]
PA01 *pelG*::Tn Δ*pslAB*	Tet^R, Gm^R, PA01 containing the WFPA60 Δ*pslAB* cassette, with the *phoA*-Tn5 transposon inserted into *pelG*	this study
Δ*yfiR cupA*::Tn	Tet^R, PA01 with the lacZ-Tn5 transposon inserted into *cupA4*	this study
Δ*yfiR cupB*::Tn	Tet^R, PA01 with the phoA-Tn5 transposon inserted into *cupB4*	this study
Δ*yfiR cupC*::Tn	Tet^R, PA01 with the lacZ-Tn5 transposon inserted into *cupC2*	this study
Δ*yfiR pelG*::Tn	Tet^R, PA01 with the phoA-Tn5 transposon inserted into *pelG*	this study
Δ*yfiR* Δ*pslAB*	Gm^R, PA01 containing the WFPA60 Δ*pslAB* cassette	this study
Δ*yfiR pelG*::Tn Δ*pslAB*	Tet^R, Gm^R, PA01 containing the WFPA60 Δ*pslAB* cassette, with the phoA-Tn5 transposon inserted into *pelG*	this study
ClinSCV-110	SCV strain isolated from CF sputum	this study
E. coli		
BL21-(DE3)	Sm^R, K12 *recF143* *lacI^q* *lacZΔ.M15*, *xylA*	Novagen
DH5α	*endA*1, *hsdR*17(r_K-m_K+), *supE*44, *recA*1, *gyrA* (Nal^r), *relA*1, Δ(*lacIZYA-argF*)U169, *deoR*, Φ80*dlacΔ(lacZ)M15*	[4]
MM337	K-12 *araD139 flbB5301* ptsF25 rbsR relA1 rpsL150 -(argF-lac)U169 -cya	M. Manson
DY330	W3110 *ΔlacU169 gal490 λcI857 Δ(cro-bioA)*	[5]
Plasmids		
pALMAR3	Insertion vector for Tet^R Mariner transposon	A. Levi
pBV-MCS4	Gm^R, vanillate inducible high-copy number plasmid	[6]
pBV-PA5295	pBV-MCS4 with PA5295 under the control of vanillate promoter	[7]
pBV-$PA5295_{E328A}$	pBV-MCS4 with $PA5292_{E328A}$ under the control of vanillate promoter	[7]
pET42b	Km^R, purification vector, His_6-tag	Novagen
pET42b-*yfiN*	pET42b with N-terminal truncated *yfiN* as *Xho*I-*Nde*I fragment	this study
pET42b-*yfiB*	pET42b with *yfiB* as *Xho*I-*Nde*I fragment	this study
pUC18T-mini-Tn7T-Gm	Amp^R, Gm^R, Tn7 insertion vector	[8]
yfiBNR-complementation	pUC18T-mini-Tn7T-Gm with *yfiBNR* operon alleles as *HindIII- BamHI*	this study

vectors	fragments. Twelve plasmids in total	
pTNS2	Amp^R, helper plasmid for Tn7 integration events	[8]
pME6032	Tet^R, P_K, 9.8 kb pVS1 derived shuttle vector	[9]
pME6032-*yfiB*	pME6032 with *yfiB* as *EcoRI-BamHI* fragment	this study
pBBR-MCS4	Amp^R, broad-host range cloning vector	[10]
pBBR-MCS5	Gm^R, broad-host range cloning vector	[10]
p-*yfiR*	pBBR-MCS5 with *yfiR* as *BamHI-HindIII* fragment	this study
pBBR4-*wspR19*	pBBR-MCS4 with *wspR19* as *EcoRI-HindIII* fragment	this study
pME-*araC*	pME6032 with *araC/pBAD*-promoter as *EcoRI-BamHI* fragment	this study
p-*ara-yfiN*	pME-*araC* with *yfiN* as *SacI -BglII* fragment	this study
pME-*araC-yfiR-phoA*	pME-*araC* with *yfiR-phoA* fusion as *EcoRI-XhoI* fragment	this study
pME-*araC*-tr-*yfiR-phoA*	pME-*araC* with trunc *yfiR-phoA* fusion as *EcoRI-XhoI* fragment	this study
pME-*yfiR-cherry*	pME6032 with *yfiR-Mcherry* fusion as *EcoRI-BglII* fragment	this study
pMR20	Tet^R, low copy number and broad host-range vector	[11]
pMR20-*yfiBNR*	pMR20 with *yfiBNR* as *HindIII-BamHI* fragment	this study
pMR20-*yfiR-M2*	pMR20 with *yfiR-M2* as *HindIII-BamHI* fragment	this study
pTn7-*yfiR-M2*	pUC18T-mini-Tn7T with *yfiR-M2* as *HindIII-BamHI* fragment	this study
pAD6Ω	Sm^R, Tet^R, pMR20 derivative containing EGFP under the *lac* promoter	this study
pKT25	pSU40 derivative with T25 fragment of CyaA	[12]
pKT25-zip	pKT25 derivative with leucine zipper of GCN4	[12]
pUT18C-zip	pUT18C derivative with leucine zipper of GCN4	[12]
pUT18C	pUC19 derivative with T18 fragment of CyaA. C-terminal fusions	[12]
pUT18	pUC19 derivative with T18 fragment of CyaA. N-terminal fusions	[12]
pUT18C-*yfi* vectors	Bacterial two-hybrid vectors with *yfi* genes as *XbaI-BamHI* fragments	this study
pKT25-*yfi* vectors	Bacterial two-hybrid vectors with *yfi* genes as *XbaI-BamHI* fragments	this study
pUT18-*yfi* vectors	Bacterial two-hybrid vectors with *yfi* genes as *HindIII-BamHI* fragments	this study
pME6032-*luxCDABE*	pME6032 with *luxCDABE* as *EcoRI-BamHI* fragment	this study
pME6032-promoter-*lux* vectors	pME6032-*luxCDABE* with PA01 promoters as *XhoI-EcoRI* fragments. Eight plasmids in total	this study
pEX18Ap	Amp^R, *oriT+*, *sacB+*, gene replacement vector	[13]
pEX18Ap-Δ*yfiBNR*	pEX18Ap with *yfiBNR* Gm^R-FRT deletion cassette as *HindIII-KpnI* fragment	this study
pEX18Ap-Δ*yfiNR*	pEX18Ap with *yfiNR* Gm^R-FRT deletion cassette as *HindIII-KpnI* fragment	this study
pEX18Ap-Δ*yfiR*	pEX18Ap with *yfiR* deletion cassette as *HindIII-KpnI* fragment	this study
pFLP2	Amp^R, FRT cassette excision vector	[13]

References

1. Holloway BW (1955) Genetic recombination in *Pseudomonas aeruginosa*. J Gen Microbiol 13: 572-581.
2. Jacobs MA, Alwood A, Thaipisuttikul I, Spencer D, Haugen E, et al. (2003) Comprehensive transposon mutant library of *Pseudomonas aeruginosa*. PNAS 100: 14339-14344.
3. Jackson KD, Starkey M, Kremer S, Parsek MR, Wozniak DJ (2004) Identification of *psl*, a locus encoding a potential exopolysaccharide that is essential for *Pseudomonas aeruginosa* PAO1 biofilm formation. J Bacteriol 186: 4466-4475.
4. Woodcock DM, Crowther PJ, Doherty J, Jefferson S, DeCruz E, et al. (1989) Quantitative evaluation of *Escherichia coli* host strains for tolerance to cytosine methylation in plasmid and phage recombinants. Nucleic Acids Res 17: 3469-3478.
5. Yu D, Ellis HM, Lee EC, Jenkins NA, Copeland NG, et al. (2000) An efficient recombination system for chromosome engineering in *Escherichia coli*. Proc Natl Acad Sci U S A 97: 5978-5983.
6. Thanbichler M, Iniesta AA, Shapiro L (2007) A comprehensive set of plasmids for vanillate- and xylose-inducible gene expression in *Caulobacter crescentus*. Nucleic Acids Res 35: e137.
7. Duerig A, Abel S, Folcher M, Nicollier M, Schwede T, et al. (2009) Second messenger-mediated spatiotemporal control of protein degradation regulates bacterial cell cycle progression. Genes Dev 23: 93-104.
8. Choi KH, Gaynor JB, White KG, Lopez C, Bosio CM, et al. (2005) A Tn*7*-based broad-range bacterial cloning and expression system. Nat Methods 2: 443-448.
9. Heeb S, Itoh Y, Nishijyo T, Schnider U, Keel C, et al. (2000) Small, stable shuttle vectors based on the minimal pVS1 replicon for use in gram-negative, plant-associated bacteria. Mol Plant Microbe Interact 13: 232-237.
10. Kovach ME, Elzer PH, Hill DS, Robertson GT, Farris MA, et al. (1995) Four new derivatives of the broad-host-range cloning vector pBBR1MCS, carrying different antibiotic-resistance cassettes. Gene 166: 175-176.
11. Roberts RC, Mohr CD, Shapiro L (1996) Developmental programs in bacteria. Curr Top Dev Biol 34: 207-257.
12. Karimova G, Ullmann A, Ladant D (2001) Protein-protein interaction between *Bacillus stearothermophilus* tyrosyl-tRNA synthetase subdomains revealed by a bacterial two-hybrid system. J Mol Microbiol Biotechnol 3: 73-82.
13. Hoang TT, Karkhoff-Schweizer RR, Kutchma AJ, Schweizer HP (1998) A broad-host-range Flp-FRT recombination system for site-specific excision of chromosomally-located DNA sequences: application for isolation of unmarked *Pseudomonas aeruginosa* mutants. Gene 212: 77-86.
