# Supplementary material for: YfiBNR Mediates Cyclic di-GMP Dependent Small Colony Variant Formation and Persistence in Pseudomonas aeruginosa
Source: PLoS Pathog. 2010 Mar 12;6(3):e1000804. doi: 10.1371/journal.ppat.1000804 (PMC2837407; doi:10.1371/journal.ppat.1000804)
Supplement: Table S2 — Primers used in this study (0.02 MB PDF) [file ppat.1000804.s002.pdf]

Table S2: Primers

Primer	*Description*	*Sequence*
A	yfiR for	CCGAAGCTTGCCGCCCCCTG
B	yfiR rev	GAGGATCCATCACGGAGTGGC
C	yfiN for	CAGAGCTCATGAACCGTCGTCGTC
D	yfiN rev	GCGGATCCTCTACTTGGGTGG
E	yfiB rev	GAGGATCCGATTACTCGGCG
F	yfiR-phoA for	CTCTCTCAATTGTTGCCTTCGTTGCCCAC
G	truncyfiR-phoA for	CTCTCTCAATTGTTGGACGCACGGACGTCGATC
H	yfiR-phoA rev	GTCTCGAGCGGAGTGGCCTGC
I	phoA for	CCACTCCGCTCGAGACCCGGACACCAGAAATG
J	phoA rev	GTGGTACCTTATTTCAGCCCCAG
K	A-site for	CTGGGCGGCGCAGAATTCGCCGTC
L	A-site rev	GACGGCGAATTCTGCGCCGCCCAG
M	I-site for	GTGGGCAGTTGGCAGAAAGCGACC
N	I-site rev	GGTCGCTTTCTGCCAACTGCCCAC
O	yfiB for	GAGAATTCGTGCTACCCCAG
P	M2lambdafor	CCCGAGCGTATTGAAACTGGCAAGGCGGCAGGCCACTCCGGACTACAAAGACCATGACGG
Q	M2lambdarev	TTACTCGGCGGGAACGATGATGGCGACGCGGCGGTTCTCGCATATGAATATCCTCCTTAG
R	omegafor	GGGGGAAAGCCACGTTGTGTCTCAAAATCTCTGATGTTACGTCATCCACCGGATCAATTC
S	omegarev	ATTCATATCAGGATTATCAATACCATATTTTTGAAAAAGCAATGCGGATGTTGCGATTAC
T	purNfor	GGCATATGCGCGGTATCGTCG
U	purNrev	GCCTCGAGCTTGGGTGGAGC
V	purBfor	GGCATATGCTACCCCAGAGGCTC
W	purBrev	GTCTCGAGCTCGGCGGGAACGATG
X	BTH-NyfiR	ACTCTAGAGGACGCACGGACGTC
Y	BTH-CyfiR F	CCAAGCTTGGACGCACGGACGTC
Z	BTH-CyfiR R	GGGGATCCTCCGGAGTGGCCTG
AA	BTH-NyfiN	ACTCTAGAGCGCGGTATCGTCG
AB	BTH-CyfiN F	CCAAGCTTGCGCGGTATCGTCG
AC	BTH-CyfiN R	GGGGATCCTCCTTGGGTGGAGC
AD	B2H-HAMP N	GGGGATCCTCTACGCCTGCCAGGATTC
AE	B2H-HAMP-C	GGGGATCCTCCGCCTGCCAGGATTC
AF	B2H-GGDEF N	ACTCTAGAGCTGCAGGACGAGAAC
AG	B2H-GGDEF C	CCAAGCTTGCTGCAGGACGAGAAC
AH	B2H-Nperi F	ACTCTAGAGGACCCCAACCAGCAAC
AI	B2H-Nperi R	GGGGATCCTCTAGTCGTGCAGGATCGG
AJ	B2H-Cperi F	CCAAGCTTGGACCCCAACCAGCAAC
AK	B2H-Cperi R	GGGGATCCTCGTCGTGCAGGATCGG
AL	psl prom F	CCACTCGAGAGATCTTGGCCGAGCGGCCTG
AM	cupB prom F	CCACTCGAGAGATCTGCTACTGGGGCAATG
AN	cupC prom F	CCACTCGAGAGATCTGAAGTCTTAGATGAG
AO	yfi prom F	CCACTCGAGAGATCTAGGATTTCCCGGATC
AP	psl prom R	CCAGAATTCGTTGTTTGCTCTG

AQ	cupB prom R	CCAGAATTCGATTGAGCTTCCTTTTG
AR	cupC prom R	CCAGAATTCCTGATTTCCTTTGGAG
AS	yfi prom R	CCAGAATTCAAAGGTTTCCTCCAG
AT	ALMAR3PCR	CGCAAACCAACCCTTGGCAG
AU	ALMAR3seq	ACATATCCATCGCGTCCGCC
AV	Arb-PCR1	GGCCAGCGAGCTAACGAGACNNNNGTTGC
AW	Arb-PCR2	GGCCAGCGAGCTAACGAGAC
AX	Gent for	CGAATTAGCTTCAAAAGCGCTCTGA
AY	Gent rev	CGAATTGGGGATCTTGAAGTTCCT
AZ	yfiBNR Up for	CCGGTACCGCTACCCGCGCATGGAACCCTTC
BA	yfiBNR Up rev	TCAGAGCGCTTTTGAAGCTAATTCGAAAGGTTTCCTCCAGGAAAAC
BB	yfiBNR Dn for	AGGAACTTCAAGATCCCCAATTCGTCCTGCGCTCGCGGGCTGC
BC	yfiBNR Dn rev	CCAAGCTTGCTAGAACGCGCAGAGCATGGC
BD	yfiNR Dn for	AGGAACTTCAAGATCCCCAATTCGTTCAGGAAGAAAAGGAG
BE	yfiNR Dn rev	CCAAGCTTGCTAGGCCGGAAGCGGGGTAGTG
BF	yfiR Up for	CCAAGCTTGCTACCCGCGCATGGAACCCTTC
BG	yfiR Up rev	CCTTGCCAGGGTGCGCCTGTAGAGGTC
BH	yfiR Dn for	AGGCGCACCCTGGCAAGGCGGCAGGCCAC
BI	yfiR Dn rev	CCGGTACCGCTCGATGCCGGCTTCCGGTAC
BJ	yfiR Cherry For	GTCTCGAGGTGAGCAAGGGCGAG
BK	yfiR Cherry rev	TAGGATCCCTTACTTGTACAGCTC
