# Supplementary material for: YfiBNR Mediates Cyclic di-GMP Dependent Small Colony Variant Formation and Persistence in Pseudomonas aeruginosa
Source: PLoS Pathog. 2010 Mar 12;6(3):e1000804. doi: 10.1371/journal.ppat.1000804 (PMC2837407; doi:10.1371/journal.ppat.1000804)
Supplement: Table S3 — Bacterial-two-hybrid results (0.02 MB PDF) [file ppat.1000804.s003.pdf]

Table S3: Bacterial two-hybrid results

No.	*N-terminal T25*	*N-terminal T18*	*C-terminal T18*	*Interaction?*
1	Leucine Zipper	Leucine Zipper		+
2	None		None	-
3	None		YfiN-truncated	-
4	None	YfiN-truncated		-
5	None		HAMP domain	-
6	None	HAMP domain		-
7	None		GGDEF domain	-
8	None	GGDEF domain		-
9	None		YfiR-truncated	-
10	None	YfiR-truncated		-
11	None	YfiN-periplasmic domain		-
12	None		YfiN-periplasmic domain	-
13	YfiN-truncated		None	-
14	YfiN-truncated		YfiN-truncated	+
15	YfiN-truncated	YfiN-truncated		+
16	YfiN-truncated		HAMP domain	+
17	YfiN-truncated	HAMP domain		-
18	YfiN-truncated		GGDEF domain	-
19	YfiN-truncated	GGDEF domain		-
20	HAMP domain		None	-
21	HAMP domain		YfiN-truncated	+
22	HAMP domain	YfiN-truncated		-
23	HAMP domain		HAMP domain	-
24	HAMP domain	HAMP domain		-
25	HAMP domain		GGDEF domain	-
26	HAMP domain	GGDEF domain		-
27	GGDEF domain		None	-
28	GGDEF domain		YfiN-truncated	-
29	GGDEF domain	YfiN-truncated		-
30	GGDEF domain		HAMP domain	-
31	GGDEF domain	HAMP domain		-
32	GGDEF domain		GGDEF domain	-
33	GGDEF domain	GGDEF domain		-
34	YfiR-truncated		None	-
35	YfiR-truncated		YfiR-truncated	-
36	YfiR-truncated	YfiR-truncated		+
37	YfiN-truncated		YfiR-truncated	-
38	YfiN-truncated	YfiR-truncated		-
39	YfiR-truncated		YfiN-truncated	-
40	YfiR-truncated	YfiN-truncated		-
41	YfiR-truncated	YfiN-periplasmic domain		-
42	YfiR-truncated		YfiN-periplasmic domain	-
