# Supplementary material for: YfiBNR Mediates Cyclic di-GMP Dependent Small Colony Variant Formation and Persistence in Pseudomonas aeruginosa
Source: PLoS Pathog. 2010 Mar 12;6(3):e1000804. doi: 10.1371/journal.ppat.1000804 (PMC2837407; doi:10.1371/journal.ppat.1000804)
Supplement: Figure S1 — Motility of the ΔyfiR and exopolysaccharide mutant strains. A) Swimming, swarming and twitching motility of Pel and Psl exopolysaccharide mutants in a wild type PA01 background. B) Motility in the ΔyfiR mutant background. Motility is abolished in the ΔyfiR mutation (5), but swimming and twitching are partially restored by disruption of Pel and Psl production (6-8). C) PA01 motility (1, 9) is unaffected by expression of yfiR in trans (10). D) The motility defect of the ΔyfiR mutant (5, 11) is restored by expression of yfiR in cis (13) or in trans (12). (0.10 MB PDF) [file ppat.1000804.s004.pdf]

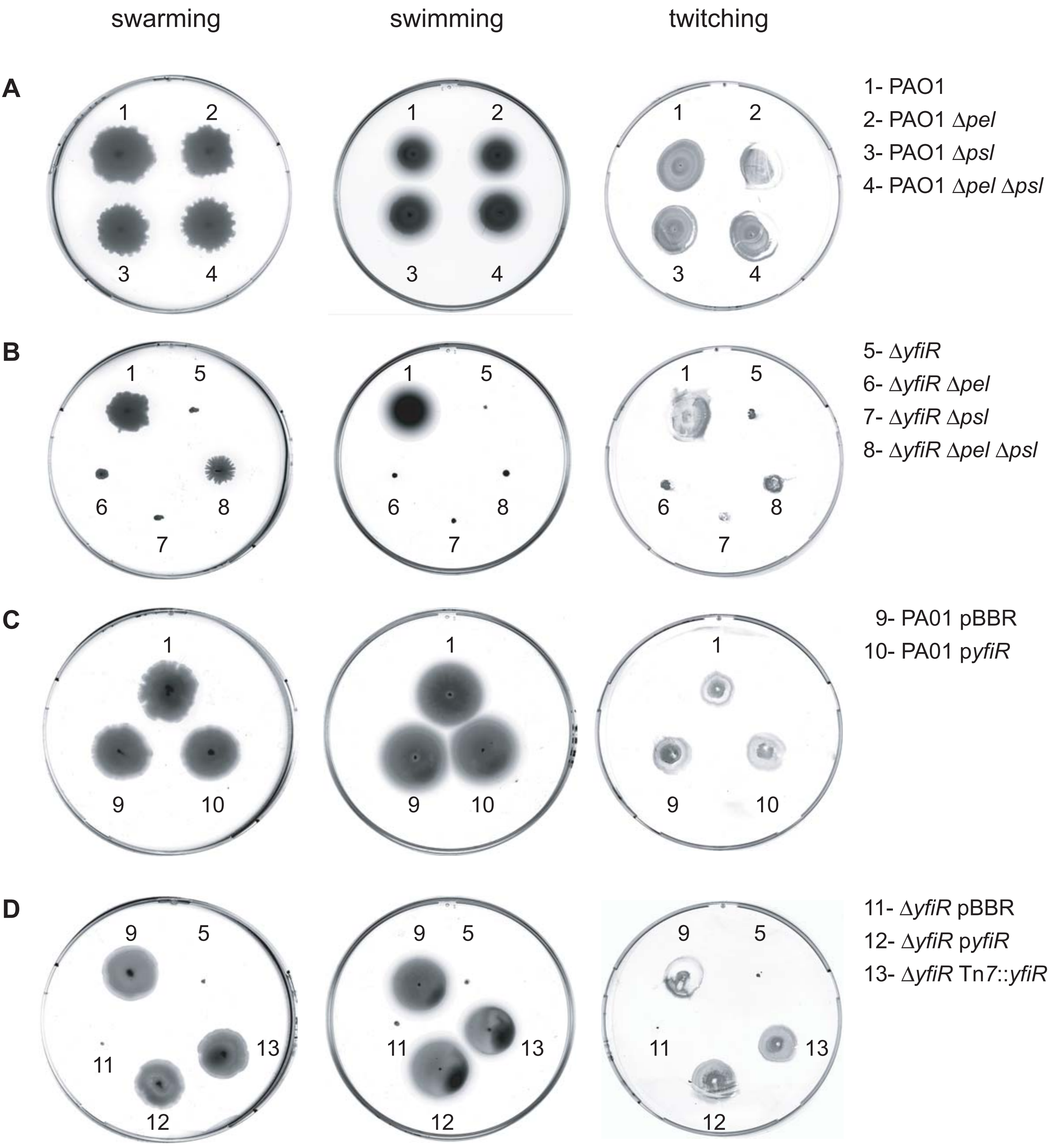
swarming
swimming
twitching
A
B
C
D
1- PAO1
2- PAO1 Δpel
3- PAO1 Δpsl
4- PAO1 Δpel Δpsl
5- ΔyfiR
6- ΔyfiR Δpel
7- ΔyfiR Δpsl
8- ΔyfiR Δpel Δpsl
9- PA01 pBBR
10- PA01 pyfiR
11- ΔyfiR pBBR
12- ΔyfiR pyfiR
13- ΔyfiR Tn7::yfiR
