# Supplementary figures and images for: YfiBNR Mediates Cyclic di-GMP Dependent Small Colony Variant Formation and Persistence in Pseudomonas aeruginosa
Source: PLoS Pathog. 2010 Mar 12;6(3):e1000804. doi: 10.1371/journal.ppat.1000804 (PMC2837407; doi:10.1371/journal.ppat.1000804)

**A**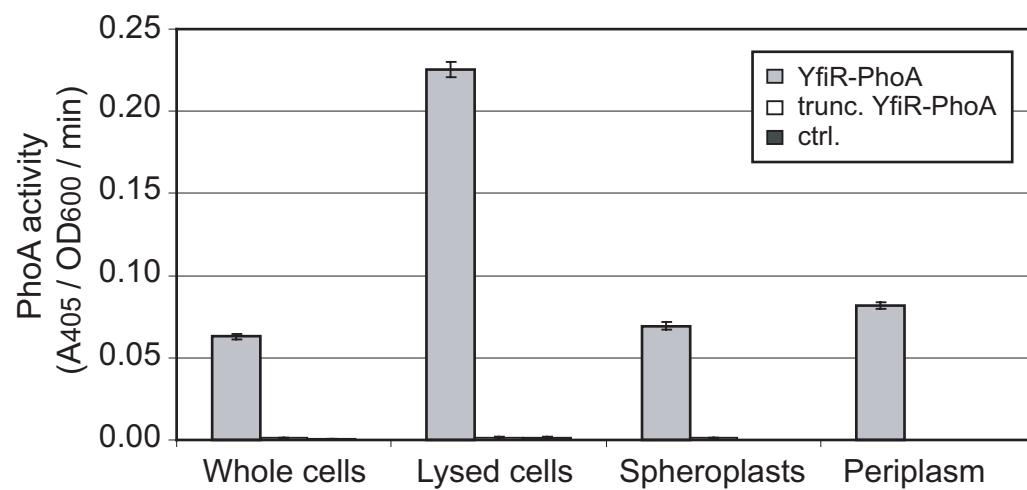**B**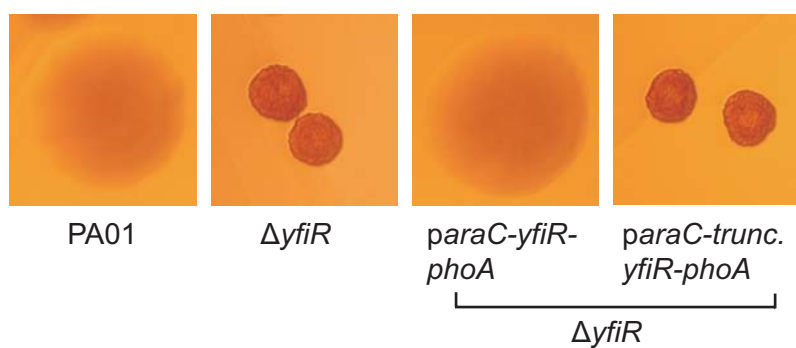**C**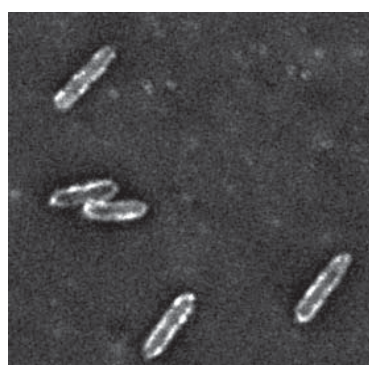

PA01 pME-*yfiR-Mcherry*

Supplement: Figure S2 — YfiR localizes to the periplasm. A) Alkaline phosphatase activity in whole-cell, spheroplast and periplasmic fractions is shown for PA01 strains expressing yfiR-phoA fusions. Activity is seen in all fractions with full-length YfiR-PhoA, but not with a truncated YfiR allele missing the first 33 residues including the signal sequence. An empty vector is used for the control. Values are expressed as A405/OD600/min ± standard error. B) Colony morphology of ΔyfiR mutants expressing yfiR-phoA fusions. Full-length YfiR-PhoA successfully complements the SCV phenotype of the ΔyfiR strain, while truncated YfiR-PhoA does not. C) YfiR-MCherry localizes to the periplasm, as determined by fluorescence microscopy. (0.04 MB PDF) [file ppat.1000804.s005.pdf]

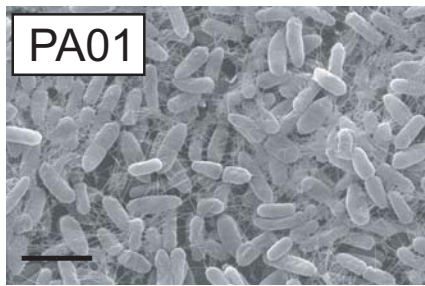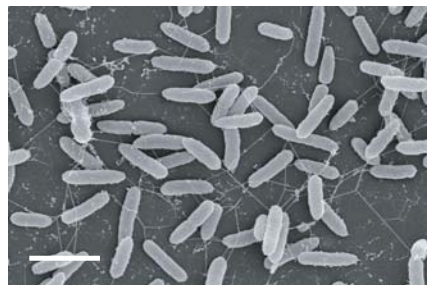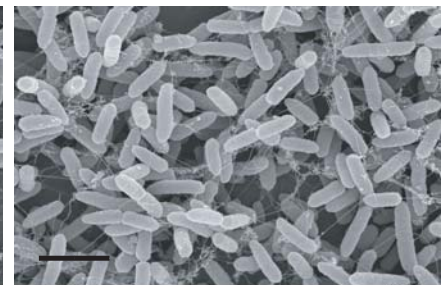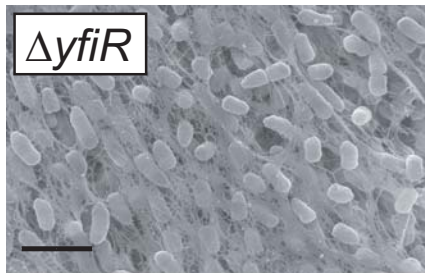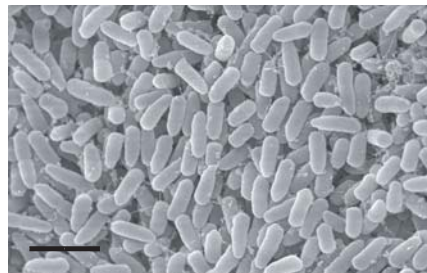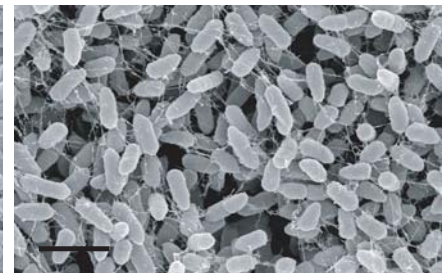

Supplement: Figure S3 — Scanning electron micrographs of PA01 and ΔyfiR exopolysaccharide mutants. The scale bars in each panel represent 2 µm. (0.12 MB PDF) [file ppat.1000804.s006.pdf]

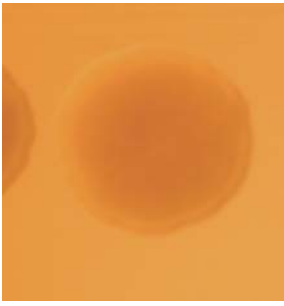

PA01

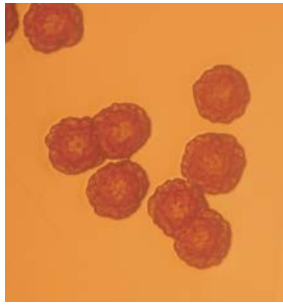

$\Delta yfiR$

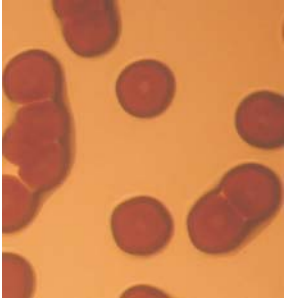

ClinSCV-110  
pMR20

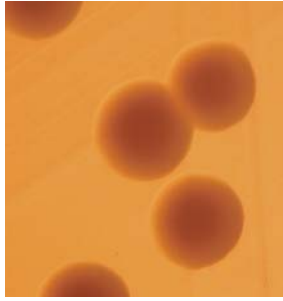

ClinSCV-110  
pMR20-*yfiR*-M2

Supplement: Figure S4 — A clinically derived SCV responds to yfiR expression. Expression of yfiR-M2 from pMR20 reverts the autoaggregative, Congo Red binding phenotypes of ClinSCV-110. PA01 and ΔyfiR strains are shown for comparison. (0.03 MB PDF) [file ppat.1000804.s007.pdf]

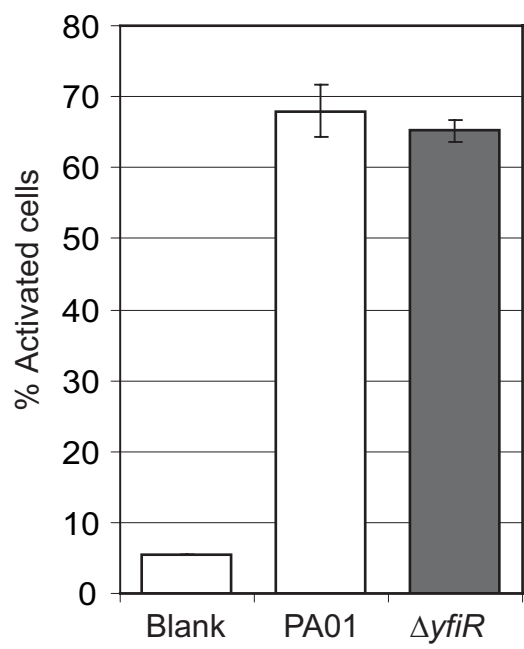

Supplement: Figure S5 — NF-κB activation by macrophages incubated with ΔyfiR and wild type PA01. NF-κB activation level in J774 macrophages was unchanged between the ΔyfiR mutant and wild type PA01. The control lane shows activation levels for macrophages incubated without bacteria. Values are expressed as the percentage of cells showing NF-κB translocation to the nucleus ± standard error. (0.01 MB PDF) [file ppat.1000804.s008.pdf]

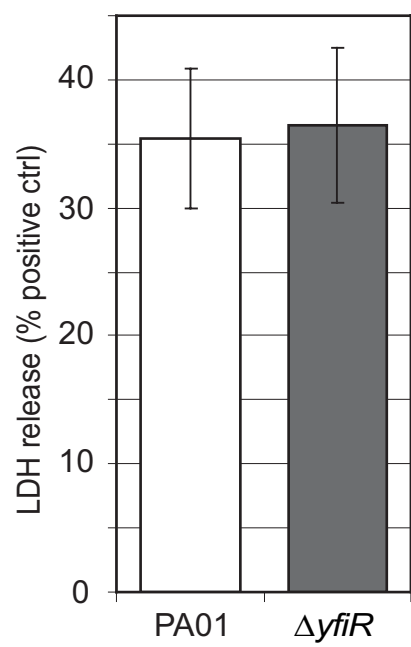

Supplement: Figure S6 — Cytotoxicity of the ΔyfiR mutant strain. No significant differences in LDH release from J774 macrophages were seen between the ΔyfiR SCV mutant and wild type PA01 under the conditions tested. Values are expressed as a percentage of the LDH released from a fully-lysed positive control sample. The graph shows the combined results of three independent experiments ± standard deviation. (0.01 MB PDF) [file ppat.1000804.s009.pdf]

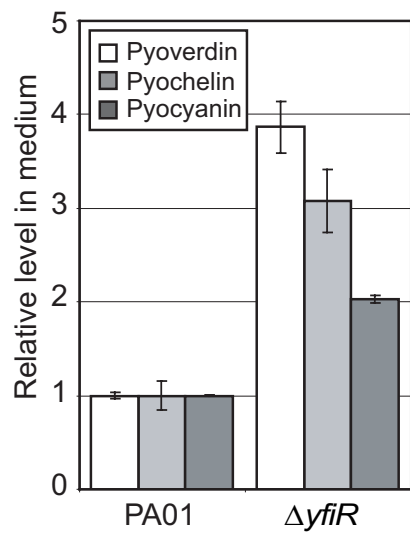

Supplement: Figure S7 — Pyocyanin and siderophore production by the ΔyfiR strain. Values are shown relative to PA01 wild type, ± standard error. (0.01 MB PDF) [file ppat.1000804.s010.pdf]

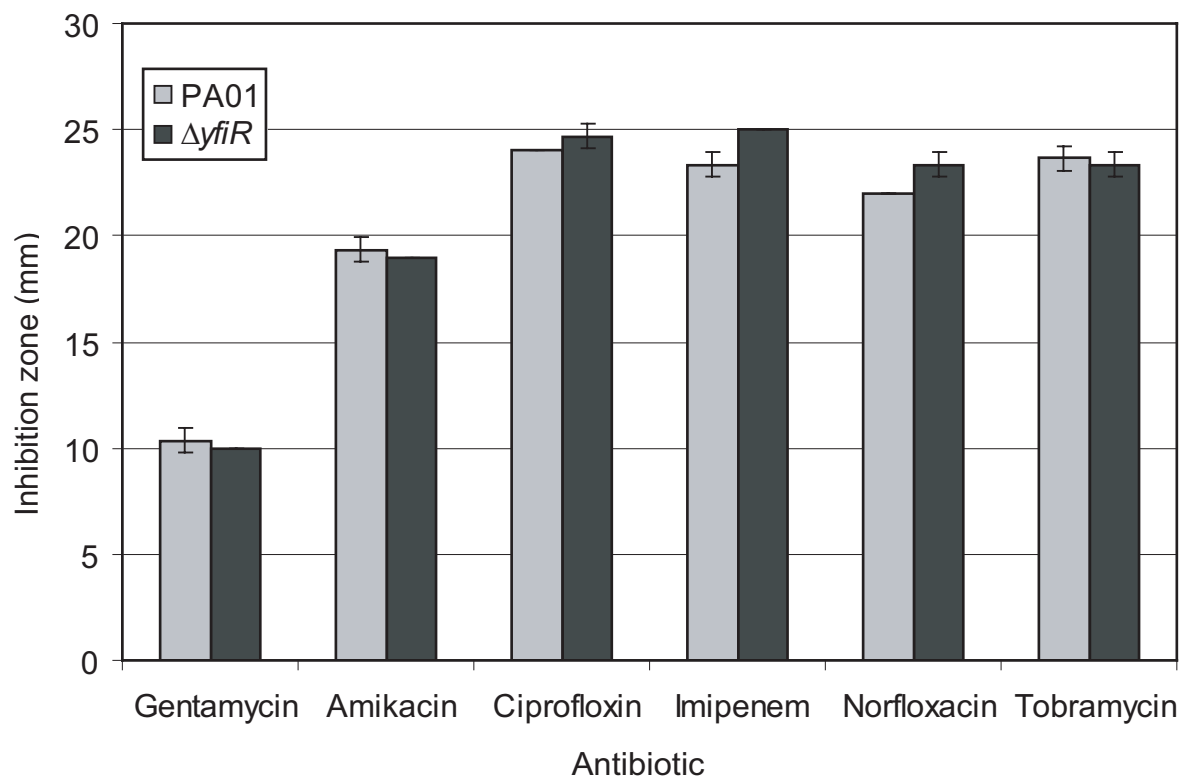

Supplement: Figure S8 — Antibiotic susceptibility of the ΔyfiR mutant strain. Inhibition zones for different antibiotic discs are shown for PA01 and the ΔyfiR mutant strains. Values shown are for the diameter of the inhibition zone in mm, and show the mean of three samples ± standard error in each case. (0.02 MB PDF) [file ppat.1000804.s011.pdf]
